# A century of exercise physiology: lung fluid balance during and following exercise

**DOI:** 10.1007/s00421-022-05066-3

**Published:** 2022-10-20

**Authors:** Giuseppe Miserocchi, Egidio Beretta

**Affiliations:** Dipartimento di Medicina e Chirurgia, Università Milano-Bicocca, Via Cadore 48, 20900 Monza, Italy

**Keywords:** Lung edema, Interstitial matrix, Interstitial pressure, Alveolar folding/unfolding, Lung diffusion, Ventilation/perfusion mismatch, Alveolar-capillary equilibration, Precapillary vasoconstriction, Pulmonary hypertension, Pulmonary arterial pressure

## Abstract

**Purpose:**

This review recalls the principles developed over a century to describe trans-capillary fluid exchanges concerning in particular the lung during exercise, a specific condition where dyspnea is a leading symptom, the question being whether this symptom simply relates to fatigue or also implies some degree of lung edema.

**Method:**

Data from experimental models of lung edema are recalled aiming to: (1) describe how extravascular lung water is strictly controlled by “safety factors” in physiological conditions, (2) consider how waning of “safety factors” inevitably leads to development of lung edema, (3) correlate data from experimental models with data from exercising humans.

**Results:**

Exercise is a strong edemagenic condition as the increase in cardiac output leads to lung capillary recruitment, increase in capillary surface for fluid exchange and potential increase in capillary pressure. The physiological low microvascular permeability may be impaired by conditions causing damage to the interstitial matrix macromolecular assembly leading to alveolar edema and haemorrhage. These conditions include hypoxia, cyclic alveolar unfolding/folding during hyperventilation putting a tensile stress on septa, intensity and duration of exercise as well as inter-individual proneness to develop lung edema.

**Conclusion:**

Data from exercising humans showed inter-individual differences in the dispersion of the lung ventilation/perfusion ratio and increase in oxygen alveolar-capillary gradient. More recent data in humans support the hypothesis that greater vasoconstriction, pulmonary hypertension and slower kinetics of alveolar-capillary O2 equilibration relate with greater proneness to develop lung edema due higher inborn microvascular permeability possibly reflecting the morpho-functional features of the air–blood barrier.

**Supplementary Information:**

The online version contains supplementary material available at 10.1007/s00421-022-05066-3.

## Introduction

The control of lung fluid balance is crucial to maintain functional gas exchange. Exercise is a specific edemagenic condition where dyspnea is a leading symptom; the question is to assess whether this symptom simply relates to fatigue or also implies some degree of developing lung edema. Further, it is well known that pulmonary hypertension develops during exercise in healthy subjects as well as patients with pulmonary and cardiovascular disease. In the latter, guidelines for the treatment of symptomatic pulmonary hypertension are based upon the degree of exercise tolerance/limitation. The first part of review describes the physiopathology of different kinds of lung edema as based on experimental models, further it provides a functional interpretation to the pulmonary hypertension that characterizes the reflex vascular response to edemagenic condition. The second part of the review aims to integrate knowledge from experimental approach with observational studies in healthy humans performing exercise. Evidence is also presented for inter-individual differences in the proneness to develop lung edema by considering inborn morpho-functional features of the air–blood barrier.

## The basic theory, its development and the necessary elements to develop knowledge

### 1893–1940. The first approach to trans-capillary fluid-exchanges

The story of interstitial fluid balance dates back to the pioneering work of Starling and Tubby (1894) and Starling (1896) concerning the clearance of fluid from serous cavities. At the time, transcapillary fluid exchanges were considered to depend upon the difference between capillary hydrostatic (*P*_C_) and colloidosmotic (Π_C_) pressures. Accordingly, the “*Starling gradient*” was given by:

1 $$\Delta P = P_{{\text{C}}} -\Pi_{{\text{c}}} .$$

Based on this concept, the idea was put forward that the longitudinal distribution of pressure in a capillary would cause fluid filtration at the arterial side and reabsorption at its venous end (Landis 1934). Shortly afterward, Danielli (1940) introduced the concept of permeability in relation to the development of edema.

### Reformulation of the Starling balance of forces

Agostoni et al. (1957) found that the visceral pleura is capable of absorbing fluid against negative pressures down to ~ − 8 mm Hg in the open chest and assumed that this value reflected the imbalance between pulmonary capillary hydraulic and colloid osmotic pressure, a conclusion still based on pressure values existing in the capillary, thus Δ$P = P_{{\text{c}}} - \Pi_{{\text{c}}}$.

The Starling model went to a profound revision by the work of Kedem and Katchalsky (1958) who considered the all set of variables existing in two compartments separated by a membrane; as an example the capillary and the interstitial compartment separated by the endothelium, or the interstitial and the alveolar compartment separated by the epithelium. The reformulation of the “*Starling gradient*” considered the hydraulic and the colloidosmotic pressures in the two compartments (identified as 1 and 2) and a factor *σ*, named reflection coefficient, expressing the permselectivity of the membrane to plasma proteins. The new formulation defines trans-membrane water flow across two adjacent compartments (*J*_v_), by introducing also the filtration coefficient *K*_f_ = *L*_p_*∙A*, being *L*_p_ the hydraulic conductance and *A* the capillary surface area available for fluid exchange:

2 $$J_{{\text{v}}} = K_{{\text{f}}} \left( {P_{1} - P_{2} } \right) - \sigma \left( {\Pi_{1} - \Pi_{2} } \right).$$

The value of *σ* varies between 0 and 1 and is a function of the molecular sieve of membrane pores relative to the size of the proteins. From experiments on the whole lung, endothelial *σ* values were found of 0.65 and 0.5 for total proteins and albumin, respectively (Taylor and Parker 1985).

On the whole, the Starling equation is perfectly still valid on theoretical ground; the challenge has always been that of estimating the values of all the terms appearing in the equation on changing functional conditions. The key point to describe transmembrane water fluxes remains that of having a sound estimate of the *Starling gradient.*

Remarkable contribution were provided to describe the morpho-functional features of the air-blood barrier, representing the interface for gas exchanges at capillary level (Weibel and Knight 1964; Weibel 1973). Further, indications concerning permeability came from the description of a “*gel like, fuzzy substance, the glycocalyx, that may play an important role in the regulation of endothelial trans-vascular flows*” (Ito 1969). For extended historical references, the reader is referred to Weinbaum et al. (2007) and to Jin et al. (2021).

### An important step: Starling dependent fluid reabsorption cannot be uphold

Contrary to Landis hypothesis, fluid reabsorption at the venous end of lung capillaries could not be uphold; in fact, reabsorption was found to be a self-limited mechanism due to the increase of the colloidosmotic pressure at the level of the glycocalyx generated by protein accumulation on the interstitial side, thus preventing further absorption (Michel and Phillips 1987). Around the same years, the role of lymphatics was being considered as the main mechanism to balance the microvascular filtration (Unruh et al. 1984; Mitzner and Sylvester 1986). Further, based on the experimental data, a model of pleural fluid turnover was developed based on Starling dependent fluid filtration and a tight control operated by pleural lymphatics, so as to maintain the pressure of the liquid in the sub-atmospheric range, thus close to a minimum volume of pleural fluid (Miserocchi et al. 1993b). The key point was to demonstrate that lymphatics are able to generate an absorption pressure in the sub-atmospheric range (Miserocchi et al. 1989).

### First attempt to estimate the pulmonary interstitial pressure

Guyton’s group estimated the pulmonary interstitial pressure via perforated capsules implanted in a lung lobe and kept in place up to 8 weeks (Meyer et al. 1968); as the authors recognized, data were affected by “*operative trauma of the lungs and infection and inflammation in and around the implanted capsules”*. Despite these limitations, values were recorded ranging from sub-atmospheric up to positive immediately after the surgery, the latter values being attributed by the authors to developing lung edema. Accordingly, the clear message from these experiments was that in the intact lung interstitial pressure was sub-atmospheric, while persisting inflammation would result in positive pressures.

### Extending the knowledge of the structure of the pulmonary interstitial compartment

Interest was being focused on the macromolecular assembly of the interstitial compartment, in particular concerning the non-fibrillar component represented by molecules from the proteoglycans family (Fessler 1957). For a complete historical review refer to Comper and Laurent (1978).

The role of proteoglycans has been largely extended as these molecules play a pivotal role in control of fluid exchanges, tissue development and repair by interacting with inflammatory cells, proteases and growth factors (Hardingham and Fosang 1992; Roberts et al. 1997; Chambers and Laurent 1996; Dunsmore and Rannels 1996). Proteoglycans fill the space left free by the fibrillar component (collagen and elastic fibers) and act as link proteins through low energy non-covalent bonds. Microvascular permeability is controlled by the low molecular weight heparan-sulphate proteoglycan (HS-PG, 300–500 kDa) coating the basement membrane and by small peptidoglycans of the glycocalyx normally assuring high resistivity to water flow down the paracellular route (Reitsma et al. 2007). Large molecular weight chondroitin-sulphate proteoglycans (CS-PG > 1000 kDa) bound to hyaluronan provide rigidity to the interstitial compartment.

### The MIGET as a tool to estimate the distribution of ventilation/perfusion ratio in exercise and potential correlation with interstitial lung edema (horses and humans)

The Multiple Inert Gas Exchange Technique (MIGET) (Yokoyama and Farhi 1967) allows measuring the pulmonary exchange of a set of six different inert gases infused intravenously. Data are used to estimate the distribution of the ventilation/perfusion ratio (${\dot{V}}_{\mathrm{A}}/\dot{Q}$), the O_2_ alveolar-capillary gradient (Δ*A − a*) and the diffusion limitation. The mechanics leading to the enlargement of the alveolar-arterial oxygen pressure gradient have been extensively described by Ferretti et al (2017). Up to 1989, accumulating evidence for exercise induced ${\dot{V}}_{\mathrm{A}}/\dot{Q}$ mismatch was found in the horse and in humans, the authors stated that mechanism as well as its clinical significance remained unknown. Further, no reference to lung edema, as a possible cause, was postulated (Wagner et al. 1989). Reference to interstitial lung edema as a potential cause of ${\dot{V}}_{\mathrm{A}}/\dot{Q}$ mismatch during maximal exercise was made by Schaffartzik et al. (1992). Further, Burnham et al. (2009) state that spatial perfusion and ${\dot{V}}_{\mathrm{A}}/\dot{Q}$ heterogeneity in heavy exercise in humans are “*consistent with but not proof of interstitial pulmonary edema*”.

### Pulmonary interstitial pressure, the key variable to define the Starling balance of forces at the level of the air-blood barrier. Data from the experimental model

**Fig. 1:**
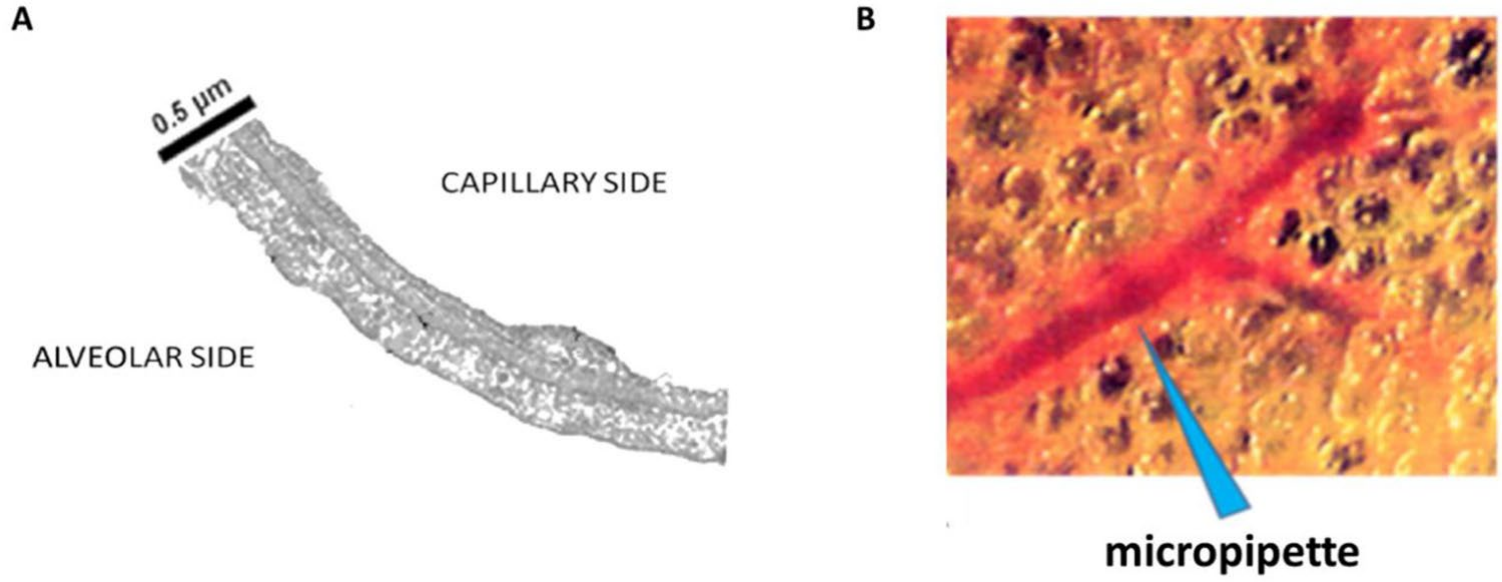
**A** Morphology of the thin portion of air-blood barrier (from Conforti et al. 2002). **B** Trans-pleural imaging of pulmonary microvessels and drawing of micropipette puncturing the peri-microvascular space

The pioneering work of Meyer et al. (1968) stimulated further research, in particular because the key variable to estimate the transendothelial and transepithelial Starling gradients was the value of the interstitial hydraulic pressure (*P*_*i*_). The obvious experimental difficulty was represented by the subtlety of the air-blood barrier, as shown in Fig. 1A. It was considered that a good compromise would be to measure the pressure in the peri-microvascular interstitial space, using micropipettes with a tip bevelled down to 2–3 µm (Fig. 1B) (Miserocchi et al. 1990). The important point of the experimental approach was that to maintain the physiological condition of the lung expanded in the chest thus exposed to a transpulmonary pressure with zero alveolar pressure (the physiological condition indeed). Micropipettes, advanced through the pleura provided a sub-atmospheric pressure of ~ – 10 cmH_2_O at the end-expiratory volume (Miserocchi et al. 1990) (Supplementary Conceptual diagram 1).

**Fig. 2:**
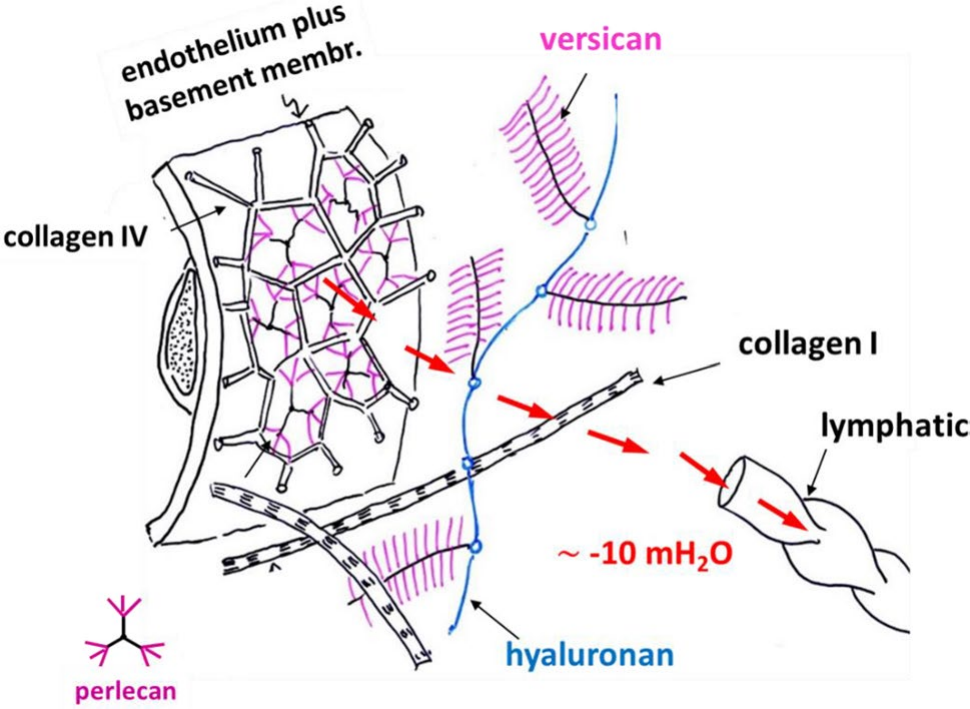
Schematic representation of the capillary wall and of the interstitial lung matrix showing its macromolecular assembly. Hydraulic interstitial pressure is also shown. Fluid filtration is balanced by lymphatic drainage (from Miserocchi and Rivolta 2012)

Figure 2 is a schematic simplified picture of the macromolecular assembly filling the interstitial space: one can identify the fibrillary collagen I, the laminar collagen IV coating the capillary surface and the molecules of perlecan (heparan sulfate proteoglycan) filling the voids of the collagen IV, then hyaluronan, a long molecule snaking through the interstitium bound at several points with versican (chondroitin sulfate proteoglycan). Versican has a core protein to which many glycans are attached. The hyaluronan-versican complex is highly hydrophilic and can easily bind free water to form gel.

Knowledge of the colloidosmotic pressure in the blood and in the interstitial space (Negrini et al. 2001), on the assumption of a reasonable value for *σ,* allowed an estimate of the trans-endothelial and the trans-epithelial Starling gradients. These data are reported in Table 1 for end-expiration and end-inspiration (30 and 70% of total lung capacity): note that trans-capillary Starling gradient is always positive (favoring capillary filtration), while trans-epithelial is always negative (favoring alveolar reabsorption).

**Table 1:** Trans-endothelial and trans-epithelial Starling pressure gradients at end-expiration (FRC) and at end-inspiration, in physiological condition (referring to Point A in Fig. 3)

	Trans-endothelial			Trans-epithelial	
	End-expiration	End-inspiration		End-expiration	End-inspiration
*P*_c_	9	9	*P*_i_	− 10	− 24
*P*_i_	− 10	− 24	*Pliq alv*	~ 0	~ 0
*σ endo*	0.85	0.85	*σ epi*	0.85	0.85
Π_c_	26.8	26.8	Π_*i*_	13.8	13.8
Π_*i*_	13.8	13.8	Π*liq alv*	0	0
			*γ*	1	1
Starling gradient	8.0	22.0	Starling gradient	− 21.7	− 35.7

Table reports the expected values for capillary, interstitial and alveolar liquid hydraulic pressure (*P*_c_, *P*_*i*_ and *Pliq alv*, respectively) as well as for capillary, interstitial and alveolar liquid oncotic pressure (*Π*_c_, *Π*_*i*_ and *Πliq alv*, respectively). Endothelial (*σ endo*) and epithelial (*σ epi*) protein reflection coefficients are also reported. In bold, the total Starling pressure gradient. Positive values of the Starling gradient at endothelial level indicate filtration into interstitium; negative value at epithelial level indicate alveolar reabsorption. Pressure values are expressed in cmH_2_O; *σ* is a pure number. Surface tension *γ* = 1 dyne/cm. From Beretta et al. (2021).

**Fig. 3:**
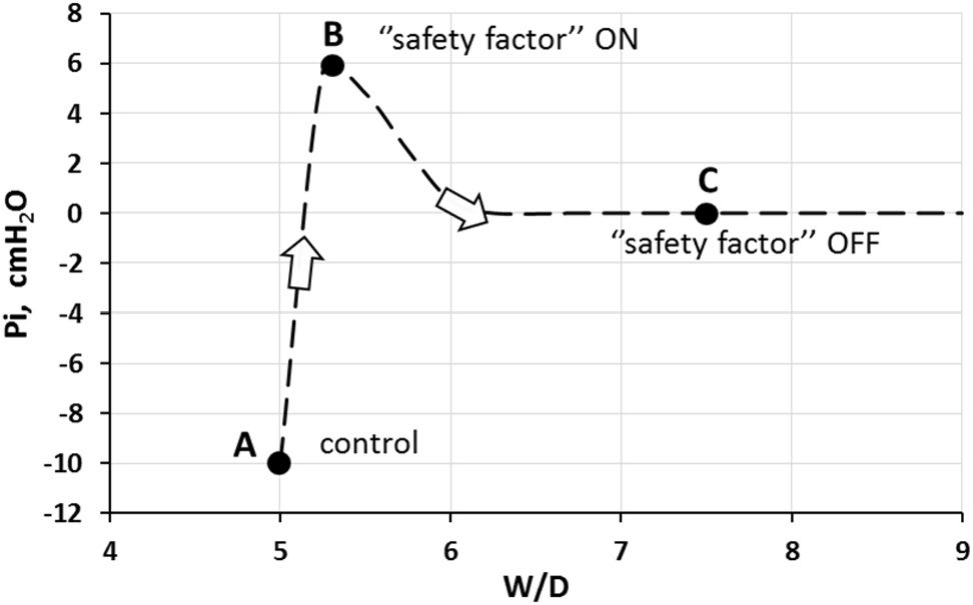
Relationship between interstitial hydraulic pressure (*P*_i_) and *W/D* ratio at end-expiration. Point A corresponds to physiological condition; point B to the “*safety factor*” (*see* text for explanation); point C corresponds to the loss of the “*safety factor*” corresponding to fragmentation of the interstitial matrix (From Beretta et al. 2021)

At interstitial level, the fluid dynamic equilibrium is set at a subatmospheric interstitial pressure resulting from the balance between capillary filtration down a high resistance pathway (low endothelial permeability) and a low resistance draining pathway represented by lymphatic drainage (Fig. 2). So, the model being proposed for the control of the extravascular water in the lung is the same as the one for the pleural space, both compartments being kept at subatmospheric pressure with a minimal water volume (Miserocchi et al. 1990; Miserocchi et al. 2001b; Miserocchi 2009).

## The pulmonary response to edemagenic condition

### The “*safety factors*” against development of edema

Concerning the microvascular district, a functional compartmentation exists between an *“extra-alveolar*” compartment and a peripheral “*true alveolar*” compartment, that are placed in series (Parker 2007). The former has a lower surface area but higher *L*_p_, it allows fluid accumulation in the prerivascular and peribronchial cuffs in edemagenic conditions and is served by an extended lymphatic network. The “*true alveolar*” compartment is designed to favour gas diffusion: besides the thinness of the air-blood barrier (see Fig. 1A), the *L*_p_ is very low and *σ* is as high as 0.97; these features minimize fluid exchange. The low permeability coefficients are considered as “*safety factors”* against development of edema. Despite an incredibly very extended surface, the “*true alveolar*” compartment is not served by an extended lymphatic network.

One shall now consider a further “*safety factor*” residing in the mechanical response of the lung interstitial matrix in developing edema. The micropuncture technique allowed to monitor the change in interstitial pressure in edemagenic conditions; three models were developed: saline loading, hypoxia exposure and a lesional model induced by injection of proteases (Miserocchi et al. 1993a, 2001a; Negrini et al. 1996).

Figure 3 shows that pulmonary interstitial pressure (*P*_*i*_) increases remarkably on increasing the wet weight to dry weight ratio (*W/D*) of the lung by about 10% in edemagenic conditions (point B), relative to control (point A). *W/D* was considered a precise and reliable index of the lung fluid balance, as shared also by Parker and Townsley (2004). What happens is that increased free water filtering in the interstitium is captured by the hyaluronan-versican complex to form gel, whose increase in steric hindrance causes a remarkable increase in *P*_*i*_ (from − 10 cmH_2_O up to ~  + 5 cmH_2_O (Miserocchi et al. 1993a; Miserocchi et al. 2001a; Negrini et al. 1996). The increase in *P*_*i*_ compared to the small increase in *W/D* ratio reflects the low tissue compliance (~ 0.5 ml·mmHg^−1^·100 g of wet weight^−1^) (Miserocchi et al. 1993a). Therefore, the increase in *P*_*i*_ nullifies the Starling filtration gradient: so a further *“safety factor”* is operating opposing the progression of edema (Supplementary Conceptual diagram 2). Shifting towards point C is discussed in the next paragraph.

### The aggravation in development of lung edema: matrix fragmentation and loss of “*safety factors*”

A sustained edemagenic condition is a cause of progressive loss of integrity of the matrix (Fig. 3C). This was demonstrated by the increase in proteoglycans fragments recovered from the lung tissue using a weak extraction agent; this technique allowed to prove the weakening of the non-covalent intermolecular bonds of degraded proteoglycans with the other components of the matrix.

**Fig. 4:**
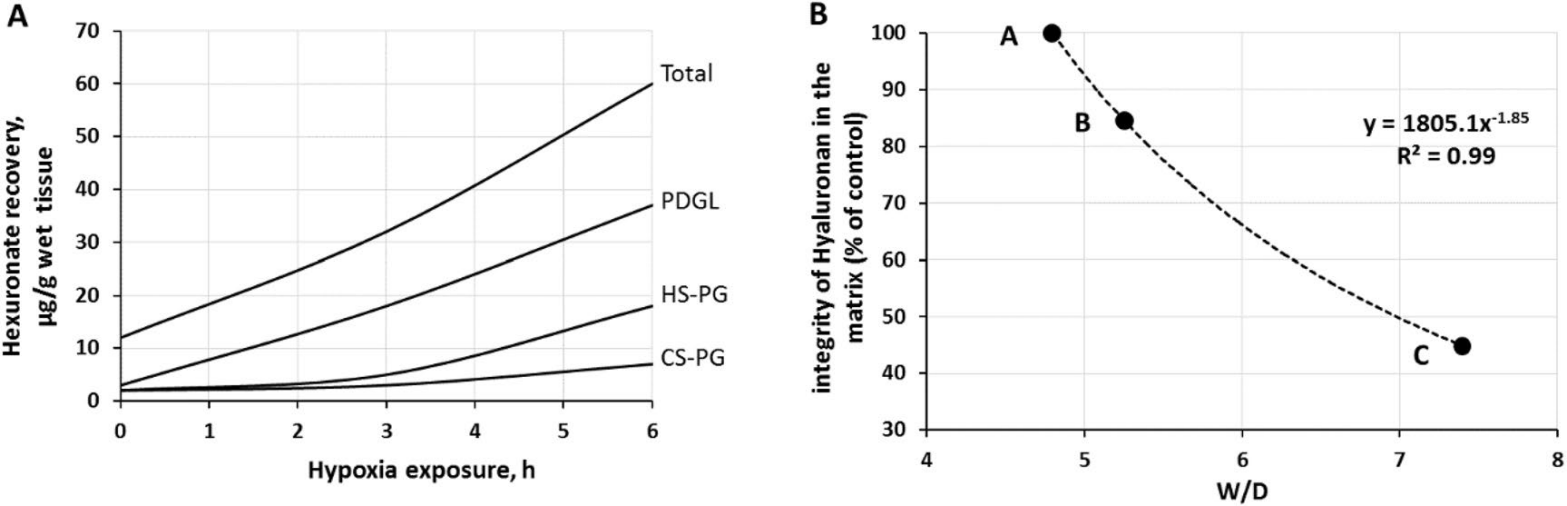
**A** Time course of the recovery of fragments of proteoglycan families on 12% O_2_ exposure (redrawn from Miserocchi et al. 2001a). PDGL (peptidoglycans) control the permeability of the glycocalyx; HS-PG (heparin-sulphate proteoglycans) control the permeability of endothelial and epithelial cells; CS-PG (chondroitin-sulphate proteoglycans) provide rigidity to the interstitial matrix. **B**: relationship between loss of integrity of interstitial hyaluronan (a hydrophilic non sulphated glycosaminoglycan) and the increase in *W/D* ratio. Points A, B, C correspond to those reported in Fig. 3 (from Beretta et al. 2021)

Figure 4A shows, as an example, the time course of the recovery of fragments of proteoglycan families on 12% O_2_ exposure (Miserocchi et al. 2001a). Figure 4B shows the negative correlation between the loss of integrity of hyaluronan and *W/D* ratio.

Differences were found in the sequence of matrix fragmentation among various models of experimental pulmonary edema: saline loading (Negrini et al. 1996), exposure to hypoxia (Miserocchi et al. 2001a) and a lesional model injecting proteases (Negrini et al. 1998; Passi et al. 1999). It was estimated that the decline in the force of the non-covalent bonds occurs when the inter-fibrillar distance exceeds 30 nm (Conforti et al. 2002).

The critical phase of aggravation of edema pivots around a *W/D* of ~ 6 to 6.5. It was hypothesized that for *W/D* exceeding 6, capillary filtration occurs by the concomitance of three factors (Supplementary Conceptual diagram 3):

- Fragmentation of large proteoglycans of the matrix results in increase in tissue compliance and consequent drop of *P*_*i*_ to zero that restores the filtration pressure gradient (Fig. 3, point C);
- Fragmentation of intermediate and small proteoglycans coating the capillary surface leads to progressive increase in microvascular permeability and decrease in *σ*;
- Possibly, a progressively larger portion of alveolar surface area is being involved in the damage.

It can be promptly understood that an increase in *Kf* (filtration coefficient) occurs through a multiplicative effect due to the increase of both *L*_p_ and surface area for filtration (see Eq. 2): these events occurring at the level of the “*true alveolar*” compartment lead to the accelerated phase of lung edema.

A recent study reported an increase in plasma HS-PG in subjects affected by high altitude lung edema after climbing up to 4558 m (Swenson et al. 2020). Although it was impossible to specify where HS-PG came from, it could not be excluded that part would come from partial degradation of the pulmonary matrix. This finding would confirm the one reported twenty years ago (Miserocchi et al. 2001a).

### Modelling capillary flow in developing edema

The micropuncture technique also allowed to describe the lung microvascular pressure profile in physiological conditions (normoxia) (Negrini et al. 1992) and during developing lung edema (Negrini et al. 2001).

**Fig. 5:**
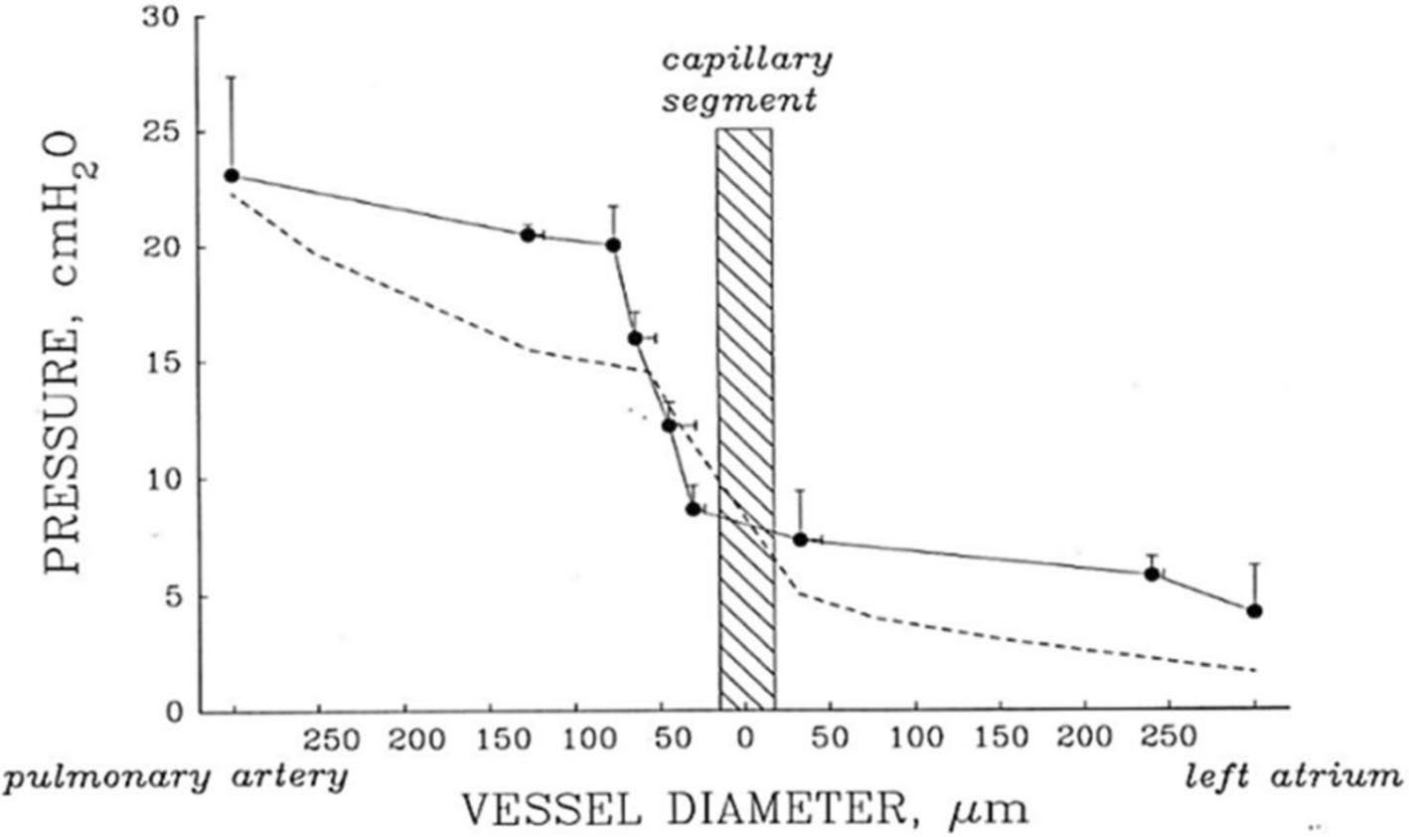
Pulmonary pressure profile from pulmonary artery to left atrium. Capillary segment corresponds to vessels of diameter ≤ 20 µm. Dashed line: physiological condition. Solid line: date referring to saline loading edema (from Negrini 1995)

The dashed line in Fig. 5 shows the drop in pressure occurring at the level of the capillaries reflecting the arteriolar precapillary resistance in control conditions (normoxia). The continuous line (closed symbols) shows how the microvascular pressure profile was modified in the edemagenic model (saline infusion) (Negrini 1995). It is clear that a further increase in precapillary resistance allows maintaining capillary pressure within the physiological value. The small increase in left atrial and post capillary pressure is compatible with a greater recruitment of the capillary network due to the increase in the circulating fluid mass.

One can now consider the role of precapillary arteriovenous shunts that are located in the “extra-alveolar” compartment. Their activation in edemagenic condition during exercise (Hopkins et al. 2009; Lovering et al. 2008, 2009; Stickland et al. 2019) would avoid an increase in alveolar capillary pressure, a further factor protecting against the development of alveolar edema. There is also evidence from direct observation of in situ lung surface with intact pleural space, that, during development of edema, vasoconstriction occurred for arterioles ~ 80 µm in diameter (Negrini et al. 2001).This vascular adjustment represents further “*safety factors*” protecting against development of edema.

Interestingly, the presence of peribronchial and perivascular edemagenic cuffs occurring in the “*extra-alveolar*” compartment have little effect on gas exchanges, while only alveolar flooding (thus the involvement of the “*true alveolar*” compartment) leads to a rapid drop in arterial PO_2_ (Taylor and Parker 1985).

**Fig. 6:**
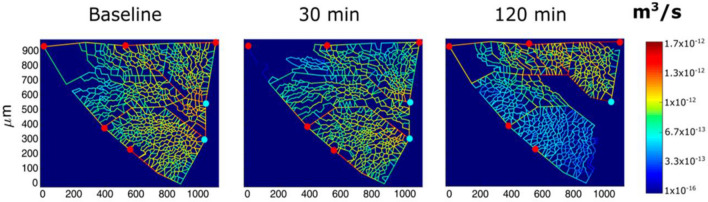
Results from modelling of alveolar perfusion in edemagenic condition (12% O_2_ exposure). Red and light blue dots identify respectively arteriolar accesses and venular exits. The color panels show capillary blood flow rates at different time points (baseline, 30, and 120 min) with color-coded log-scale intensity. In regions where edema develops capillary blood flow is progressively reduced approaching zero (from Mazzuca et al. 2019)

Edema development in the lung is patchy although favoured by gravity; noteworthy, redirection of blood flow from edematous to non edematous regions has been demonstrated (Rivolta et al. 2011). Blood flow switching among capillaries has been described as a passive mechanism reflecting the branching geometry, the transcapillary pressure gradient as well as the longitudinal profile of capillary pressure (Mazzuca et al. 2019). As an example, Fig. 6 shows the capillary perfusion pattern with a color-coded log-scale in alveolar districts after hypoxia exposure (Mazzuca et al. 2019). Progressive closure of microvessels (blue colour) was observed over time in alveolar regions where interstitial edema was more marked, as judged from the thickness of the perivascular interstitial space. Accordingly, besides precapillary vasoconstriction, capillary closure could result due to the compressive action of the increase in interstitial pressure in developing edema (Mazzuca et al. 2019). De-recruitment of alveolar capillaries may also occur on increasing lung volume simply due to increase in tensile parenchymal stretching, a phenomenon may that may be favoured by peri-microvascular edema (*see*  paragraph *Alveolar unfolding-folding*).

Microvascular filtration flow could also be modelled on changing blood flow by entering the adequate parameters (Mazzuca et al. 2016): giving the heterogeneous distribution of blood flow, it was found that the filtration flow was also heterogeneously distributed. Given the heterogeneities in capillary flow distribution, it appeared tempting to consider that blood flow diversion from regions being less resistant to edema to more resistant ones represents an intrinsic mechanism to control lung fluid balance and preserve the efficiency of the diffusion/perfusion function (Rivolta et al. 2011; Mazzuca et al. 2016, 2019).

Derecruitment due to perivascular edema may well occur also in humans, as administration of a vasodilator agent cannot restore blood flow in edematous lung regions (Scherrer et al. 1996).

An interesting point from the work of Mazzuca et al. (2019) is that lower fluid extravasation was observed around smaller alveoli. The question remains on how alveolar morphology might influence the stability of extravascular fluid control. Fractal geometry has been invoked to describe blood flow and ventilation in terminal units (Glenny 2011): the model considered that small asymmetries along the branching system might account for heterogeneity in both airways and blood flow distribution.

### The relative roles of the edemagenic factors

A comprehensive and integrated model has been developed to account for the different contribution of the various factors appearing in Eq. 2 to the developing phase of edema (Mazzuca et al. 2016). Based on the results from the different experimental models of edema, it appears that several factors might contribute to the kinetics of edema formation, although to a different extent. Yet, the kern of edema formation is the loss of the “*safety factors*” that assure in physiological conditions the mechanical resistance to fluid extravasation (low microvascular permeability) and tissue resistance to mechanical stress (low tissue compliance). The model suggests that increasing pulmonary capillary pressure (from 9 to 25 cmH_2_O) is, by and large, more edemagenic compared to an eightfold increase in water permeability (*L*_p_): the difference can be explained considering that the increase in capillary pressure contributes to capillary recruitment, so that a greater *Starling gradient* is acting on a large surface area. The same model showed a remarkable acceleration in edema formation if the increase in capillary pressure was coupled with the increase in *L*_p_*,* the specific case of exercise in hypoxia.

## Lung cellular signalling of developing edema

Given the remarkable change in interstitial pressure and the potential onset of matrix degradation in developing edema, a key question was to ascertain whether an early cellular signalling could be detected in response to the extravascular perturbation.

Changes in proteins responsible for signal transduction and cell–cell adhesion as well as in membrane proteins involved in membrane-to-cytoskeleton linkage were found in endothelial cells with a ~ 10% increase in extravascular water (*Safety factor*), both with infusion and hypoxia models of edema (Botto et al. 2006; Daffara et al. 2004).

Moreover, in A549 cells and in primary human alveolar cells exposed to hypoxia (12%O_2_), significant remodelling was observed of the plasmalemmal dynamic signalling platforms (so called lipid microdomains) (Botto et al. 2008). It was concluded that these modifications observed both in endothelial and epithelial cells in edemagenic condition provide the evidence for prompt activation of a mechano-transduction signalling process evoked by the increase of *P*_*i*_ for a minimal increase in extravascular water. Further, a differential expression of signalling platforms might actually reflect the damage to a precise components of the extracellular matrix and thus can trigger a molecule-oriented specific matrix remodelling (Palestini et al. 2011). Interestingly, endothelial and epithelial cells are highly deformed in vivo being kept in a flat shape due to their strong attachments to the neighbouring cells and to the extracellular matrix. Accordingly, their “*hardwired*” cytoskeleton allows to respond promptly to forces/pressures applied on their surface or transmitted through the cytoskeleton that plays an important role in mechano-transduction (Hamill and Martinac 2001; Ingber 2003; Morris and Homann 2001).

Gene expression analysis was carried out in a saline infusion model of edema. Cytokines TNFα and MT1 (a stress response molecule) were markedly up-regulated during the induction phase representing a pro-inflammatory response; interestingly, IFNγ, a molecule involved in increase in permeability and apoptosis, was down-regulated, a response clearly aimed at preserving the endothelial barrier. Further, MT1 genes (acting as antioxidant agents) markedly increased their expression, as well as bFGF, involved in vascular/tissue remodelling. Results suggested that stimuli affecting mRNA modulation in the endothelial cells may originate either on luminal side, due to shear stress and vascular distension, or at tissue level, due to parenchymal forces caused by matrix imbibition. The message from the study was that mechano-transduction could be considered as the first event leading to the production of cytokines, which could act as early mediators able to induce secondary effects including interstitial matrix turnover (Sabbadini et al. 2003).

**Fig. 7:**
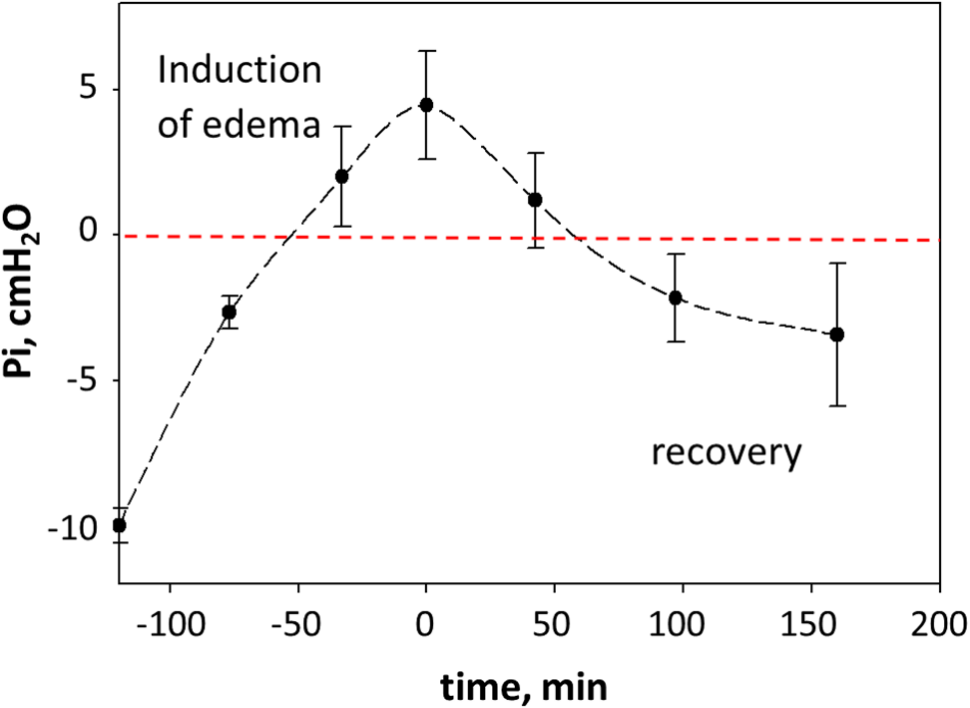
Time course of pulmonary interstitial pressure during induction of edema and after removal of the edemagenic factor: interstitial pressure shift towards negative value (unpublished data from Sabbadini et al. 2003)

Figure 7 shows the time course of *P*_*i*_ in the recovery phase from a saline loading edema model when the infusion was stopped. It can be appreciated that about 3 h were required for *P*_*i*_ to return below 0 down to ~ -– 5 cmH_2_O.

Proteoglycan remodelling is delayed relative to the signalling phase, ranging from a few hours to 0.5 day (Brown et al. 1991; Laurent and Fraser 1992).

## Physiopathological mechanisms in various types of exercise

We shall now analyse some cases of exercise causing edema where the physiopathological mechanisms differ substantially in terms of specific environmental conditions, power output and duration. An attempt will be made to identify the main edemagenic factor and/or which combination of edemagenic factors are acting in exercise induced lung edema.

### Alveolar unfolding-folding

Exercise implies a further edemagenic factor relating with hyperventilation, namely the cyclic alveolar “*unfolding-folding*” process. To explain this mechanism one shall recall the specific topology of Epithelial cells Type 1 (Epi1) (Weibel 2015). Any Epi1 cell covers about 4 endothelial cells and present numerous pleats on its surface that are subjected to the cyclic *“unfolding–folding”* process on changing lung volume (Bachofen et al. 1987). Based on data from electron microscopy, unfolding was found to be completed in healthy lung on reaching ~ 65% of vital capacity, corresponding to lung distending pressure ~15cm H_2_O.

**Fig. 8:**
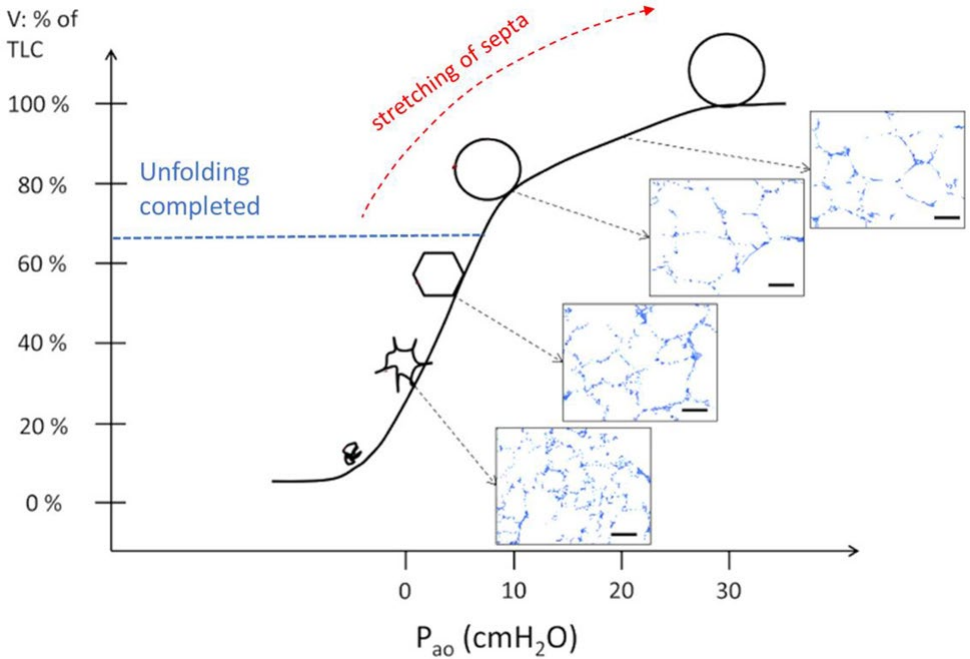
The unfolding-folding process in relation with lung volume, lung distending pressure (*Pao*) and alveolar morphology (redrawn from Knudsen and Ochs 2018)

Figure 8 describes the *unfolding–folding* process in relation with lung volume, lung distending pressure and alveolar morphology. Septa are exposed to increase in parenchymal tension on increasing lung volume and clearly, as the unfolding process is being completed, the septa become exposed to increased mechanical tissue stretching (causing a decrease in lung compliance) that may result in lesion of the alveolar structure (Knudsen and Ochs 2018). Note also, recalling data from Table 1, that at end-inspiration *P*_*i*_, due to parenchymal stretching, becomes rather sub-atmospheric (Miserocchi et al. 1991), thus potentially increasing the endothelial filtration gradient.

Other data confirm the edemagenic nature of increasing lung volume: data by Egan (1982) suggest that acute lung distension may cause epithelial albumin leak; further, increasing transpulmonary pressure to about 20 cmH_2_O over 6 h was found to cause a fivefold increase in capillary filtration (Tarbell et al. 1999; Tarbell 2010) and moreover *W/D* was found to increase well above 6 for *Palv* > 20 cmH_2_O kept for 2 h (Yoshikawa et al. 2004). The *unfolding–folding* process has been confirmed also for the human lung for a similar range of lung distending pressure (Miserocchi et al. 2008; Beretta et al. 2021).

### Capillary stress failure as an early cause of lung edema: diving in apnea

This is a pure case of stress failure of pulmonary capillaries leading to edema rapidly developing during immersion time (on the average 2–3 min). Convincing modelling strongly based on respiratory mechanics showed that transcapillary pressure gradient is remarkably increased at depth due to the large drop in alveolar air pressure, resulting from the outward elastic recoil of the chest wall undergoing compression by the increase in hydrostatic pressure at depth (Fitz-Clarke 2007a, b). An estimate of 50 mmHg of transcapillary pressure was obtained for record of immersion at 183 m. Obviously, capillary stress failure entails haemorrhage and protein leak. Diving also implies a further edemagenic factor given by the remarkable blood pooling in the lung (Arborelius et al. 1972).

### The case of thoroughbred horses

This is a case of acute lung edema developing for all-out exercise lasting about 2 min, the average racing time for a horse. Lung haemorrhage has been widely documented in competing thoroughbred horses (Whitwell and Greet 1984). Increasing power output requires an increase in oxygen delivery that is accomplished by an increase in cardiac output coupled with the increase in pulmonary oxygen uptake. The latter is favoured by lowering the precapillary arteriolar tone allowing capillary recruitment. In a horse race, 100% $\dot{V}{\text{O}}_{2\max }$ (about 250 ml/Kg/min) is attained within 15–20 s from the start through an increase in cardiac output up to about 600 ml/kg/min, and in ventilation in the range of 1500L/min; the maximal speed attained in the last 400 m is in the range of 17 m/s. At a speed of 13 m/s, pulmonary artery and capillary pressure were found to range about 90 mmHg and 75 mmHg, respectively (Manohar 1993). The high value of capillary pressure stems for a relatively lower upstream resistance allowing indeed extensive capillary recruitment. The capillary recruitment favours gas diffusion and avoids excessive shortening of the blood capillary transit time; however, the price to be paid is an increase in capillary filtration down an increased pressure gradient and increased capillary surface area. Accordingly, the likely pathophysiological interpretation on the origin of acute capillary stress failure in racing horses may well relate with the remarkable increase in capillary pressure (mechanical factor), increase in surface area and shear (permeability factors); the latter represent the adaptive response to reach $\dot{V}{\text{O}}_{2\max }$ in a very short time (Manohar 1994). Further the *unfolding-folding* might also play a substantial role given the incredibly high ventilation. The possible co-existence of an increase in *L*_p_ relating with developing hypoxemia could be considered (Wagner et al. 1989), although it is short lasting.

Maximum power output in humans at $\dot{V}{\text{O}}_{2\max }$ (such as in middle distance running, 800–1500 m) have not been reported to cause edema (Zavorsky 2007). It remains difficult to scale power output at $\dot{V}{\text{O}}_{2\max }$ from horse to humans, noting however that, unlike humans, horses are vigorously whipped!

### A doubtful case: exercise at high altitude

One shall now consider exercise at high altitude that is known to potentially cause lung edema (Swenson and Bärtsch 2012). Unlike the case of thoroughbred horses, high altitude pulmonary edema (HAPE) it is not an acute phenomenon as it develops over 2–5 days; accordingly, over this time the process of matrix degradation triggered by hypoxia should have advanced leading to a progressive increase in microvascular permeability (due to fragmentation of HS-PG), as well as weakening of the mechanical resistance of the capillary wall and surrounding macromolecular structure (due to fragmentation of CS-PG) (*see* paragraph *Lung cellular signalling of developing edema* and Fig. 6).

Pulmonary artery pressure (PAP) values have been reported in the range 40–50 mmHg with corresponding capillary pressure (from wedge pressure) of 20–25 mmHg (Maggiorini et al. 2001). Hultgren et al. (1964) reported an increase in PAP reaching about 60 mmHg in subjects developing lung edema at an altitude of 3750 m but wedge pressure never exceeded 10 mmHg, thus revealing a remarkable increase in precapillary pulmonary vascular resistance. In 1987 an exhaustive paper was published describing the functional adaptation of the pulmonary circulation in subjects maintained in a hypobaric chamber for 40 days where barometric pressure was progressively decreased down to that corresponding to the summit of Mt. Everest (240 mmHg) (Groves et al. 1987). From sea level to an ambient pressure of 240 mmHg, PAP increased at rest from $\sim$ 15 to $\sim$ 34 mmHg, while at maximal exercise PAP attained $\sim$ 34 and $\sim$ 54 mmHg, respectively. The increase precapillary arteriolar resistance allowed to maintain pulmonary wedge pressure below 10 mmHg, both at rest and during exercise. Interestingly, 100% O_2_ breathing lowered cardiac output and PAP, but did not change pulmonary vascular resistance, a finding suggesting a stable increase in pre-capillary flow resistance compatible with a mechanical constraint on capillary patency due to interstitial edema. Arteriolar vasoconstriction has been documented on hypoxia exposure in lowlanders (Dunham-Snary et al. 2017; Moudgil et al. 2005). It is of interest that chronic exposure to high altitude induces pulmonary vascular remodelling leading to fibrosis and development of sustained pulmonary hypertension, representing an increased load on the right ventricle. There is no reported evidence for development of lung edema in these subjects (Sydykov et al. 2021). One may comment that the excessive deposition of fibrotic tissue in the lung parenchyma as well as pulmonary artery fibrosis provides a form of maladaptive protection against development of lung edema, as it occurs at the expense of increasing right-ventricular afterload.

Stress failure of pulmonary capillaries has been invoked as the main cause of HAPE (West et al. 1991), by analogy with capillary stress failure in thoroughbred horses. However, concerning capillary resistance to wall mechanical stress in experiments similar to those of West, different results were obtained (Bachofen et al. 1993; Wu et al. 1995): endothelial lesions were rare in high pressure experiments (about 30 mmHg), while there were distinct and numerous lesions of the epithelial Epi1 cells. As already noted, Epi1 cells are exposed to the risk of lesion by the *unfolding-folding* mechanism characteristically accompanying the hyperventilation. One may add that when Epi1 cells are lesioned, they no longer cover the underlying network of intercellular junctions that represents a potential paracellular filtration route. This point has been so far overlooked.

In High Altitude Pulmonary Edema Sensitive (HAPE-S) subjects, a more marked vasoconstrictor response to hypoxia in the pulmonary circulation was also described in the systemic circulation, a finding attributed to impaired vascular endothelial function due decreased bioavailability of NO (Berger et al. 2005). Yet, a decrease in exhaled NO was also found in HAPE-S subjects on exposure to normobaric hypoxia (Busch et al. 2001) as well as in patients with HAPE (Duplain et al. 2000). On a causative basis, it remains to be established whether the low bioavailability of NO reflects an impairment of the biochemical pathway or, conversely, represents the functional response to counteract edema formation. Based on physio-pathological evidence, pulmonary hypertension developing during exercise may well be regarded not as the cause of a perturbation in lung fluid balance, but rather as the consequence of a protective functional response to limit edema formation (*see* PROPOSAL 1).

Concerning the hypothesis of pulmonary hypertension caused by “*excessive rise in pulmonary vascular resistance…… leading to increased microvascular pressures*” (Swenson and Bärtsch 2012), it remains counter-intuitive how a rise in pulmonary vascular resistance could lead to downstream increased microvascular pressure. So, the above hypothesis still requires the validation by a fluid dynamic model. The likely most important factors causing edema during work in exercise are the increase in microvascular permeability (increase in *L*_p_ and decrease in *σ*) and the *unfolding-folding* mechanism. As high altitude edema occurs preferentially in some individuals, one could invoke an inborn capillary fragility possibly reflecting higher microvascular permeability (*see* paragraph *The kinetics of alveolar-capillary oxygen equilibration*). It is therefore doubtful that stress failure does occur as initial acute phenomenon in high altitude edema. This point of view is shared by Hopkins who considers that “*capillary stress failure is the extreme point on the continuum of the lung’s response to stress and is not required for the development of pulmonary edema”* (Hopkins 2010b). Indeed, there may be a variable time dependent contribution to edema formation due the concomitance of changes in pressure/surface of capillaries, increase in microvascular permeability and/or increase in tissue compliance (Miserocchi et al. 2001b). It is important to note here that considering a sharp distinction between permeability and hydraulic edema represents a widely used but simplistic and possibly erroneous concept. In fact, as already noted in paragraph *Lung cellular signalling of developing edema*, the progressive increase in microvascular permeability may well occur concurrently with changes in capillary pressure/surface, although relative intensity of effects and timing may differ. This clarification might contribute to provide an answer to a puzzling point made by Swenson et al. 2002 who maintained that “*high permeability edema is not readily explained by classical hydrostatic (Starling forces), which should generate a protein poor alveolar edema”*: the comment is that the case hypothesized is a non-existing one; further, since the definition of Starling forces 64 years ago (Kedem and Katchalsky 1958), there is no conundrum in accepting that any combination of changes of the terms appearing in Eq. 2 may justify the development of lung edema.

### Ultralong performances

An experimental study in rats exposed to simulated altitude of 4700 m, forced by electrical stimulation to walk for 48 h (with 15–20 min breaks every 4 h), at a speed of 12 m/min gave the following results: 37% died before; in survivors, the lungs were highly compromised (Bai et al. 2010) showing complete rupture of the blood-gas barrier including both the endothelium and the epithelium. The authors did not find signs of inflammation and propose that stress failure plays a major role in the findings. One can share the concept of stress failure, however it should be interpreted as the consequence of a “*lung’s response to stress”* in terms of overuse: this might well include a persistent increase in capillary pressure and recruitment as well as the infinite sequence of *unfolding-folding* events during hyperventilation. Such a case could well occur in other long/ultra-long performances. Interstitial lung edema may be induced by marathon running (Zavorsky et al. 2014). There is a report of pulmonary edema after the Bicycle Race Across America (4800 km), a relatively low-intensity effort at about 50% of $\dot{V}{\text{O}}_{2\max }$ (Luks et al. 2007), while good tolerance was reported for another participant (Schumacher et al. 2011). Further there is a report for two cases of pulmonary edema in a ultra-marathon of 90 km (McKechnie et al. 1979).

## Data that are consistent with but not proof of interstitial pulmonary edema during exercise

### Functional data

Most of the evidence for developing some degree of lung edema during exercise was based on the data coming from the MIGET technique based on the dispersion of the $\dot{V}_{{\text{A}}} /\dot{Q}$ distribution. MIGET actually cannot determine the morpho-functional or mechanical causes for the impairment in gas exchange. The hypothesis is that fluid accumulation around the microvessels, in the small airways as well as in the alveoli, is the likely cause substantially contributing to $\dot{V}_{{\text{A}}} /\dot{Q}$ heterogeneity and diffusion limitation during exercise. Regional variability in the development of edema have been provided in experimental models; it appears therefore conceivable that the progressive dispersion of $\dot{V}_{{\text{A}}} /\dot{Q}$ over time might include alveolar flooding (causing a decrease in $\dot{V}_{{\text{A}}} /\dot{Q}$) and capillary blood flow limitation (causing an increase in $\dot{V}_{A} /\dot{Q})$ (Supplementary Conceptual diagram 2 and 3). Non-ventilated and non-perfused alveoli contribute to diffusion limitation. Hammond et al. (1986b) hypothesized that development of mild interstitial edema could contribute to the $\dot{V}_{{\text{A}}} /\dot{Q}$ dispersion during work in normoxia and hypoxia. Reference to interstitial lung edema as a potential cause of $\dot{V}_{{\text{A}}} /\dot{Q}$ mismatch was made by Schaffartzik et al. (1992) during maximal exercise. Log standard deviation of perfusion and ventilation distributions (Log SDQ, and log SDv, respectively) were correlated with the increase in *ΔA-aO*_*2*_ and diffusion limitation (Hopkins et al. 1998a; Hopkins 2005, 2020). More recent data confirm that 45 min at about 80% $\dot{V}{\text{O}}_{2\max }$ increased $\dot{V}_{{\text{A}}} /\dot{Q}$ mismatch in the basal lung after exercise, likely reflecting gravitationally dependent interstitial pulmonary edema (Tedjasaputra et al. 2019).

In humans it was found that exercise induces remarkable redistribution of pulmonary blood flow, in particular in the apical regions (Harf et al. 1978) possibly reflecting re-direction of blood flow away from basal regions, that are more exposed to development of edema. Heterogeneity in pulmonary perfusion in sustained heavy exercise in humans was confirmed (Burnham et al. 2009). Techniques available for measuring perfusion are dealt with in the comprehensive review by Hopkins et al. (2012).

### The high frequency oscillation technique

In the experimental model, the hypothesis was put forward to assess whether the mechanical properties of lung would be significantly affected when the lung tissue matrix is put under tension in interstitial edema (point B in Fig. 3). The problem was afforded by adopting the non-invasive “*frequency oscillation technique*” to estimate the changes in the mechanical properties of the respiratory system. This technique allows to derive the impedance of the respiratory system by oscillating it at different frequencies. Based on oscillation frequency imposed at the mouth, one can derive indications of the mechanical behaviour of the lung without the cooperation of the subject (Goldman et al. 2005). The system provides in particular: (a) the resistance at 5 Hz (R5), (b) the resistance at 20 Hz (R20), (c) the difference R5−R20, expression of the frequency dependence of resistance, (d) the reactance at 5 Hz (X5) that reflects the visco-elastic properties of the distal lung portion. A whole oscillation cycle lasts about 30 s and the manoeuver could be repeatedly performed during the experiment. Results indicated that the progressive changes in the impedenzometric indexes occurring during developing interstitial edema likely reflected the changes in the visco-elastic properties of the lung tissue in relation with the specific mechanical condition relating with the increase in extravascular water not exceeding 10% (Dellacà et al. 2008). Extending the use of the impedenzometric technique from the experimental animal to humans, one could actually confirm a greater lung water perturbation in some subjects exercising at sea level and at altitude. The result was interpreted as corresponding to a greater individual proneness to develop lung edema (Bartesaghi et al. 2014). S*ee* PROPOSAL 2.

### Ultrasound

Lung ultrasound (LUS) is a potential semiquantitative operator dependent tool for detecting lung edema, possibly discriminating between interstitial edema and alveolar edema (Picano and Pellikka 2016). The no-echo signal (black lung in physiological conditions) shifts towards a black and white pattern on increasing extravascular water by the appearance of peculiar echo fingerprints, named B-lines. LUS was used to identify signs of pulmonary edema in scuba divers (Castagna et al. 2018), as well as in swimmers over a distance between 1000 and 3000 m (Hårdstedt et al. 2020). Concerning HAPE, the work of Nowadly et al (2021) reported no LUS signs in 15 climbers during the ascent to the Mount Everest base camp (5.380 mt).

### Analysis of exhaled air

One can quote an interesting research development aiming to investigate temperature, relative humidity and water evaporation in exhaled air (Mansour et al. 2019). The results of the study define the ranges of temperature and relative humidity of exhaled breath and the correlation with clinical and environmental factors such as gender, BMI and age. These factors ought to be taken into consideration in order to increase the reproducibility and reliability of a wide variety of measurements. It was proposed that humidity in the exhaled air was significantly lower in rats developing acute pulmonary edema (induced by a-naphthylthiourea) compared to healthy rats. The result supports the hypothesis that humidity in expiratory air can be considered an important parameter to follow-up pulmonary edema in patients (Adar et al. 2022). S*ee* PROPOSAL 3.

## Data after exercise

### Functional data

A convincing demonstration that a perturbation induced by exercise takes some time to disappear is the persistence of ${\dot{V}}_{\mathrm{A}}/\dot{Q}$ inequality with increasing $\dot{V}{\mathrm{O}}_{2}$ and diffusion limitation at least transiently following exercise relative to rest with no change after giving 100% O_2_ (Hammond et al. 1986a, Podolsky et al. 1996).

### Imaging data

Sheel and McKenzie (2010) stated that “*attempts to determine the presence of pulmonary edema in humans with modern, objective imaging techniques and other methods have failed to provide supportive evidence for its existence”*.

Most of the controversies concerning the development of exercise induced edema reflect the sensitivity of the imaging methods available to detect an increase in extravascular water (Hopkins 2020).

We report a comparison of a new proton MRI technique to measure regional ${\dot{V}}_{\mathrm{A}}/\dot{Q}$ ratio against the multiple inert gas elimination technique (MIGET). The study reports good relationships between measures of heterogeneity derived from MIGET and those derived from MRI. Although currently limited to a single slice acquisition, these data suggest that single sagittal slice measures of ${\dot{V}}_{\mathrm{A}}/\dot{Q}$ ratio provide an adequate means to assess heterogeneity in the normal lung (Sá et al. 2017). One can mention a recent study allowing to define the spatial/temporal dynamics of blood flow in the human lung by MRI in subjected developing HAPE, exhibiting known differences in vascular reactivity (Buxton et al. 2022).

Other MRI data reported increased ventilation-perfusion mismatch only in the basal lung of athletes following 45 min of cycling exercise, consistent with the development of lung edema in the gravitationally dependent lung during heavy exercise (Tedjasaputra et al. 2019).

Data from CT scan also reveal development of lung edema in highly trained athletes after performing a triathlon (Caillaud et al. 1995). A shareable point is made that a likely cause of edema would be an increase in capillary permeability that is independent of high vascular pressures (Lovering 2010).

The clearance rate of aerosolized 99mTc-labeled diethylenetriaminepentaacetic acid as an index for the permeability of the lung blood-gas barrier (Hanel et al. 2003) was found to increase after maximal workout in rowing, suggesting an increase in microvascular permeability.

### Calibration of imaging data with *W/D* ratio

So far, imaging data have not been correlated with the corresponding regional values of *W/D* ratios. It would be useful to define such correlation (*see* PROPOSAL 4) to detect the earliest stages of perturbation in lung fluid balance before the condition becomes life-threatening. In particular, it would be useful to identify intrapulmonary lung regions with a *W/D* ~ 6.5 (corresponding to a 35% increase in extravascular lung water) likely representing the critical threshold for developing severe edema (Beretta et al. 2021; Cressoni et al. 2013; Negrini et al. 2001; Taylor and Parker 1985).

**Fig. 9:**
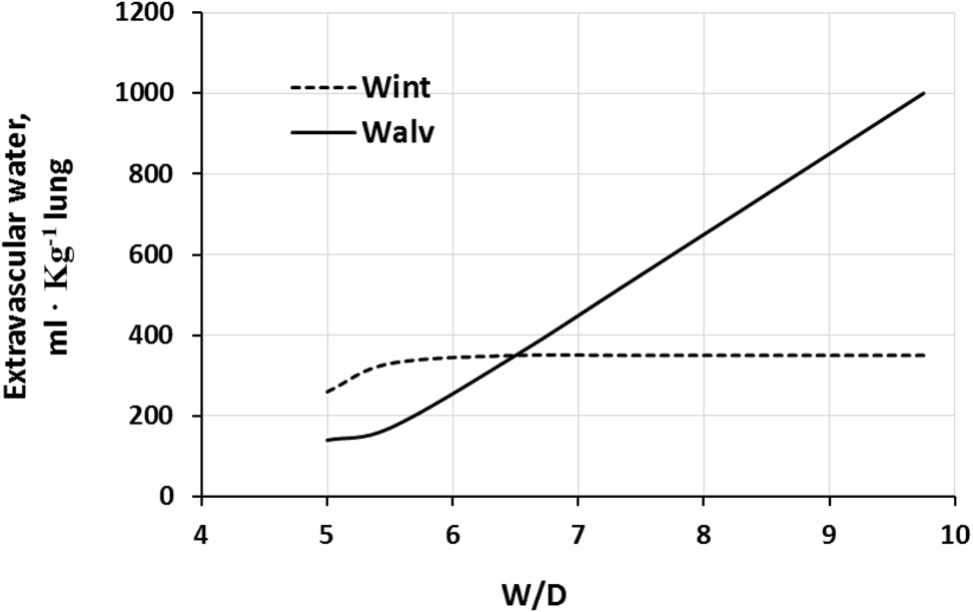
Distribution of extravascular lung water to the interstitial (Wint) and alveolar (Walv) compartment on increasing *W/D* (from Beretta et al. 2021)

Figure 9 shows that at *W/D* of ~ 6.5, the capacity of the interstitial compartment for fluid accumulation (*Wint*) is saturated while alveolar flooding (*Walv*) will continue to increase on increasing *W/D* (Beretta et al. 2021).

### Broncho-alveolar lavage fluid (BAL)

Most of the interest in estimating BAL contents pivots around finding markers of inflammation; their presence would explain the increase in permeability leading to various degree of leakyness of the capillary wall, justifying the finding of plasma proteins (in particular the largest one, albumin) and erythrocytes. One should mention that innate immunity is activated by sterile type of conditions, including tissue/vascular stretch and hypoxia that indeed represent inflammatory agents activating signalling molecules named HMGB1 (High mobility group box 1) (Andersson and Tracey 2011; Yang et al. 2020). So, one cannot exclude that this early detection system is activated during exercise and hypoxia exposure.

Potent mediators of inflammation, namely leukotriene B4 and C5a, were found in BAL on reaching 4400 m after some days of trekking together with high-molecular-weight proteins and erythrocytes. The correct biophysical interpretation was that high altitude edema involves a leak through "*large pores*" (a specific term modelling barrier permselectivity), thus a permeability type of edema reflecting an inflammatory process (Schoene et al. 1986). These results were confirmed by a following paper (Schoene et al. 1988). Increase of leukotriene B4, following maximal exercise lasting 6–8 min was also found in BAL 1 h after exercise. Further, in subjects with a history suggestive of lung edema, higher concentrations of red cells and proteins were found in BAL (Hopkins et al. 1998b).

A further report referring to cycle ergometer at 90% maximal effort in hypobaric hypoxia indicated a significantly greater leakage of red blood cells and protein into the alveolar space compared to normoxia, data extending up to 26 h after exercise. Results were interpreted as being due to progressive increase in microvascular permeability possibly evolving towards capillary stress failure (Eldridge et al. 2006).

All these data are obviously in line with the mRNA activation triggering the pre-inflammatory response to the initial phase of developing edema (Sabbadini et al. 2003).

One shall now discuss the data presented by Swenson et al. (2002) referring to post exercise ascent to Margherita Hut (4550 m). It was found that at altitude, HAPE resistant (HAPE-R) subjects had a small but significant increase in total protein and albumin in BAL (but not red blood cells), in agreement with data from Schoene et al. (1986, 1988). One can explain these findings by a progressive increase in microvascular permeability. Unfortunately, no data were provided as to the time course of the increase in albumin as well as erythrocytes in HAPE-S subjects. It is logic to consider that, as much as in the HAPE-R, the data would have shown a progressive increase, although likely reflecting a more tumultuous kinetics. The other factors reported in BAL cannot be considered as pre-inflammatory or inflammatory. Note that neutrophil accumulation in the BAL was already shown not to be characteristic in HAPE (Schoene et al. 1986). Further, uphold the stress-failure hypothesis by showing a positive correlation between the increase in pulmonary artery pressure and albumin concentration in BAL, cannot be taken as a cause-effect result, as it is simply a phenomenological process.

The conclusion for non-inflammatory process by Swenson et al. (2002) is questionable, as it neglects the progressive damage to the interstitial-capillary complex occurring on exposure to hypoxia during the time elapsing before overt edema is diagnosed. Noteworthy, the conclusion is at variance with data previously presented, providing the evidence of inflammatory response with increased permeability in HAPE (Bärtsch 1999), where Swenson was the last author.

## Edema formation in relation with power output and duration of exercise

One can report here the conclusions from the extensive review by Zavorsky (2007). It was concluded that radiographic, CT or MRI imaging seems to be equally effective in detecting pulmonary edema. A correlation was found based on intensity of exercise. Bouts of submaximal exercise as well as the incremental $\dot{V}{\text{O}}_{2\max }$ test, not exceeding 20–25 min time, are not sufficient to trigger pulmonary edema. In case of prolonged exercise at 50–75% $\dot{V}{\text{O}}_{2\max }$, ranging from 15 min up to 2 h, 16% of subjects might show signs of edema. The likelihood of developing clinical pulmonary oedema increases remarkably, reaching 65% in the category defined as MAX EFFORT. In this category, by combining intensity and duration of exercise, the subjects perceived effort was considered quite high, the general attitude being highly competitive and aimed at finish in the fastest possible time. Interestingly, the likelihood of developing pulmonary edema appeared to be independent of sex and fitness level, even on hypoxia exposure in the range of 149–106 mmHg inspired *P*O_*2*_ (about 3500 m). Actually, the persistence of ${\dot{V}}_{\mathrm{A}}/\dot{Q}$ mismatch up to 20 min after return of ventilation and cardiac output to resting values in a group of subjects after near-maximal exercise in hypoxia (inspired *P*O_*2*_ ~ 90 mmHg) reinforced the hypothesis of lung edema in a group of subjects (Schaffartzik et al. 1992). Other data from MIGET extending up to 60 min after exercise are consistent with interstitial pulmonary edema in trained high level athletes working at 70% $\dot{V}{\text{O}}_{2\max }$ for 45 min (Burnham et al. 2009).

One may comment that interstitial edema (“*safety factor*”) was likely present in all subjects during exercise and further the variability in the post-exercise determinations could be accounted for by the inter-individual differences in the proneness to progression of edema during work. One can obviously share the comment that”*the severity and prevalence of edema during exercise could be more than what’s reported post-exercise*” (Zavorsky 2007).

## Evidence for inter-individual differences in the morpho-functional features of the air–blood barrier, response to exercise and proneness to develop lung edema

Gender differences have been considered in terms of proneness to develop edema. Women might be more exposed to the risk of lung edema due to lower lung diffusing capacity, smaller airway diameter and smaller lung volumes than men (Hopkins and Harms 2004). However, available data do not support this hypothesis (Zavorsky 2007).

A large inter-individual variability of Log SDQ and Log SDV was reported since the work by Wagner et al. (1986, 1987). Moreover, two groups of subjects were in fact considered having different responses to exercise (Schaffartzik et al. 1992). Further, results indicated that in athletes with a history suggestive of lung bleeding, altered blood-gas barrier function after brief intense exercise resulted in higher concentrations of red cells and protein in BAL fluid (Hopkins et al. 1997). Further, the evidence was provided that pulmonary clearance rates of 99mTc-DTPA, a nonspecific but sensitive method for detecting lung fluid clearance, could be opposite among the subjects studied comparing rest to after exercise (Edwards et al. 2000).

In a further study extravascular water was estimated by MRI after 45 min of heavy bicycle exercise; 90 min after the end of exercise 50% of subjects showed increased extravascular water, while the remaining 50% had no change (McKenzie et al. 2005).

Differences in the proneness to develop a perturbation in lung fluid balance have been confirmed also on exposure to hypoxia by distinguishing subject being more prone to develop high altitude edema defined HAPE-S subjects (Busch et al. 2001; Canouie-Poitrine et al. 2014; Dehler et al. 2006; Eichstaedt et al. 2020; Richalet et al. 2012; Richalet et al. 2021; Hanaoka et al. 2000).

Finding reason for these differences has remained elusive for a long time. Some hints came attempting to characterize the morpho-functional features of the alveolar-capillary unit, considering the ratio of the pulmonary capillary blood volume (*V*_c_, an index of the extension of the capillary network) and the alveolar membrane diffusive property (*D*_m_) (Miserocchi et al. 2008). The distribution of the *V*_c_*/D*_m_ ratio in the subjects studied was found to be normal varying between ~ 1 up to ~ 6. During exercise at sea level and in hypoxia (3840 m), a remarkable de-recruitment of pulmonary capillaries was found in subjects having a high *V*_c_*/D*_m_ while minor-derecruitment or actually some recruitment was observed in subjects with low *V*_c_*/D*_m_ (Bartesaghi et al. 2014; Beretta et al. 2017b).

People having a low *V*_c_*/D*_m_ were shown to respond to work in hypoxia with a less marked increase in ventilation, that dampens the edemagenic effect of the *unfolding-folding* process. Since these subjects also showed a lower increase in cardiac output, maintaining however a higher O_2_ blood saturation, a low *V*_c_*/D*_m_ seems to assure a more efficient alveolar-capillary oxygen diffusion-transport (Bartesaghi et al. 2014). Subjects having a higher *V*_c_*/D*_m_ value also showed a greater perturbation in lung fluid balance during exercise, both at sea level and at altitude (Bartesaghi et al. 2014). Numerical simulations allowed to characterise a low *V*_c_*/D*_m_ as reflecting a higher number of alveoli of smaller radius providing a high alveolar surface but a limited extension of the capillary network, just opposite features for high *V*_c_*/D*_m_ value. It is challenging to consider that the group with a low *V*_c_*/D*_m_ appears to be favoured considering several aspects of the oxygen function chain are also more resistant to edema. We like to recall that “*the best possible acinus*” should be small so that the length of its perimeter is of the order of the length travelled by a gas molecule before being absorbed (Felici et al. 2003; Sapoval 1993).

## The kinetics of alveolar-capillary oxygen equilibration

The development of an oxygen *ΔA-a* during exercise may result from different mechanisms: the $\dot{V}_{{\text{A}}} /\dot{Q}$ mismatch, pulmonary diffusion limitation, shunt and decrease in transit time. We propose here the results from a novel analysis based on the development of the mass balance equation for oxygen transfer from alveoli to blood (Piiper and Scheid 1981). Defining ${\text{d}}\dot{M}$ the oxygen mass transport across the air-blood barrier, $\dot{Q}$ the cardiac output and $dC$ the increase in blood oxygen concentration along the length of the pulmonary capillary (*x*), the following equation holds, grounded on the equality between O_2_ diffusion and O_2_ transport in the blood under steady state condition:

3 $${\text{d}}\dot{M}\left( x \right) = \dot{Q} \cdot {\text{d}}C\left( x \right).$$

The mathematical development, as detailed in Beretta et al. (2019), allows to define an equilibrium (*L*_eq_) at the exit from the capillary as:

4 $$L_{{{\text{eq}}}} = \frac{{P_{{\text{A}}} - P_{{\text{a}}} }}{{P_{{\text{A}}} - P_{{\overline{v}}} }}e^{{ - \frac{{D{\text{O}}_{2} }}{{\beta \dot{Q}}}}}$$

where, *P*_A_, *P*_a_ and $P_{{\overline{v}}}$ are the partial pressures of O_2_ in the alveoli, in the arterial blood leaving the lung and in the mixed venous blood, respectively; *D*O_*2*_ is the oxygen diffusive capacity, $\dot{Q}$ is the cardiac output and *β* is the haemoglobin binding capacity for oxygen, expressing the mean slope of the Hb-O_2_ dissociation curve.

Based on the exponential kinetics of the equilibration process, one can also write:

5 $$L_{{{\text{eq}}}} = e^{{ - \frac{T{\rm t}}{\tau }}}$$

being *T*^*t*^ the average blood transit time in the pulmonary capillary that can be estimated as the ratio of the lung capillary volume (*V*_c_) to cardiac output ($\dot{Q}$*)*:

6 $$T_{{\text{t}}} = \frac{{V_{{\text{c}}} }}{{\dot{Q}}},$$

and *τ* the time constant of the equilibration process is thus defined as:

7 $$\tau = \frac{{\beta V_{{\text{c}}} }}{{D{\text{O}}_{2} }}.$$

*L*_eq_ can vary from 0 (the case of perfect equilibration) to 1 (the case of 100% shunt).

Obviously, the same *L*_eq_ value is obtained regardless of the exponent being used, either $\frac{{D{\text{O}}_{2} }}{{\beta \dot{Q}}}$ or $\frac{{T_{{\text{t}}} }}{\tau }$. These exponents provide different but complementary information concerning the functional response to the increase in oxygen demand in hypoxia. Note that *D*_m_ linearly correlates with DLCO and *D*O_*2*_ (Beretta et al. 2017a).

**Fig. 10:**
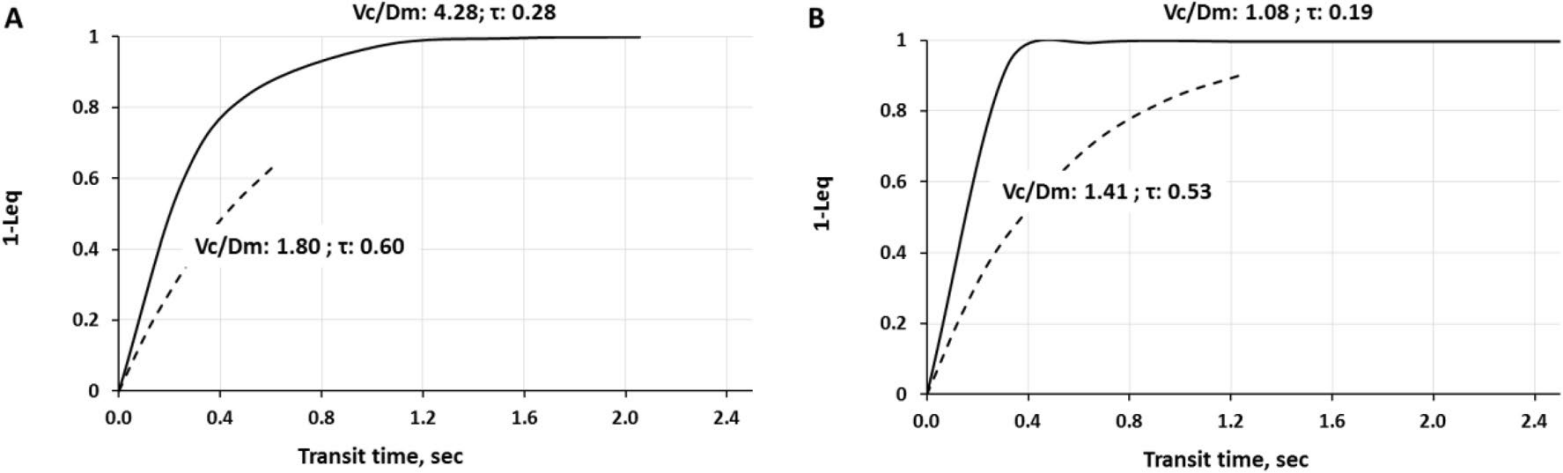
Time course of 1 − *L*_eq_ (index of alveolar-capillary equilibration) in normoxia at rest (continuous line) and on exposure to severe hypoxia (3840 m, *P*_I_O_2_ 90 mmHg, dashed line) in two representative subjects having a high (**A**) or a low (**B**) *V*_c_*/D*_m_ ratio at sea level in resting condition (from Miserocchi et al. 2022)

The continuous lines in Fig. 10 show the time course of alveolar-capillary equilibration for two representative subjects at rest in normoxia having a *V*_c_*/D*_m_ ratio of 4.28 (panel A) and 1.08 (panel B), respectively. For the sake of graphical representation we put on the ordinate 1 − *L*_eq_, meaning that the case of perfect equilibration implies *L*_eq_ = 1.

In normoxia at rest, equilibration kinetics is remarkably slower in subject with a high *V*_c_*/D*_m_ : $\tau$ is longer due to a higher *V*_c_ and a relatively low *D*O_2_; opposite features for the other subject having a lower *V*_c_*/D*_m_ . In both subjects at rest, *T*_t_ is long enough to allow complete equilibration. During work in severe hypoxia (3840 m, *P*_I_*O*_2_ 90 mmHg, dashed lines) in both subjects $\tau$ increased mostly reflecting the increase in *β*, slowing down the kinetics of equilibration.

Panel A shows that the most important factor limiting equilibration is the remarkable shortening of *T*_*t*_ due to vasoconstriction (decrease in *V*_c_*/D*_m_ from 4.28 to 1.8; Supplementary Conceptual diagram 4). In panel B equilibration was only slightly impaired as *T*_*t*_ was longer due to vasodilation (increase in *V*_c_*/D*_m_ from 1.08 to 1.41). The increase in cardiac output was similar in the two subjects. It is noteworthy that subject with a low *V*_c_*/D*_m_ had a very high *D*_m_ value also reflecting a high total lung volume for his height. In fact, a large lung volume has been considered an important determinant to favour gas exchange during exercise, despite the occurrence of interstitial edema affecting ${\dot{V}}_{\mathrm{A}}/\dot{Q}$ distribution (Hopkins et al. 1998a).

**Fig. 11:**
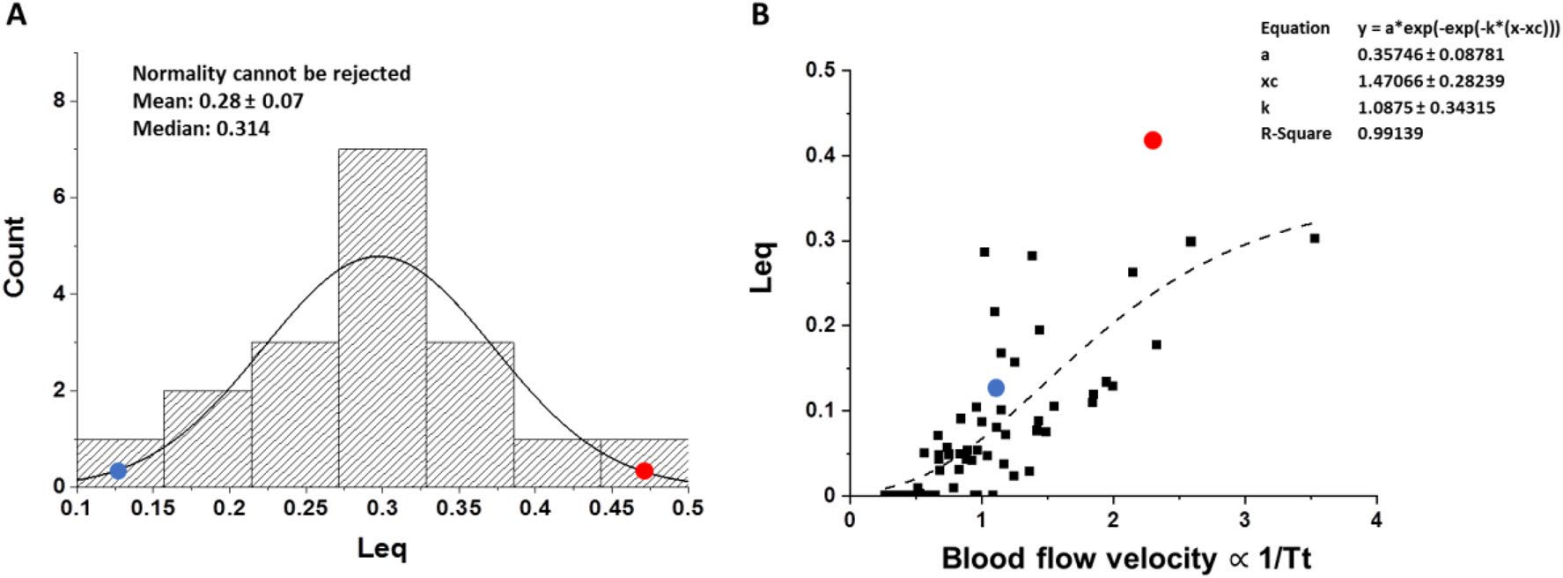
**A** Distribution of *L*_eq_ values at the end of exercise in severe hypoxia (3840 m, P_I_O_2_ 90 mmHg). Red and blue dots refer to the two subjects shown in Fig. 10A, B, respectively. **B** Plot of *L*_eq_vs 1/*T*_t_ (as an index of blood flow velocity that is proportional to shear rate). Red and blue dots refer to the two subjects shown in Fig. 10A, B, respectively (from Miserocchi et al. 2022)

Figure 11A shows that the distribution of *L*_eq_ values is normal in the group of subjects studied; the blue and red dots refer to subjects with low and high *V*_c_*/D*_m_ ratio, respectively. Figure 11B allows to estimate the correlation between *L*_eq_ and 1/*Tt*. We consider that *1/Tt* is proportional to blood velocity and hypothesize that its increase causes an increase in shear, an important factor leading to an increase in microvascular permeability (Miserocchi et al. 2022): in fact, on comparing the two subjects, *L*_eq_ increases remarkably in the red subject (higher blood velocity and shear) compared to the blue subject.

In summary, *T*_t_ reflects the interaction between the individual increase in $\dot{Q}$(required by exercise) and the available extension of the capillary network. Shortening of *T*_t_ causes a shunt-like effect. These conclusions extend previous observations by Hopkins et al. (1996).

The existence of precapillary shunts in exercise allows the following consideration: shunted blood will contribute to widen the oxygen *ΔA-a*; since the flow reaching capillaries is lower than total cardiac output, the decrease in transit time is actually less than expected relative to the one calculated from $\frac{{V_{{\text{c}}} }}{{\dot{Q}}}$ ratio; further, shunting limits the increase in capillary pressure (Eldridge et al. 2004). Modelling gas diffusion/transport together with microvascular fluid dynamics at alveolar-capillary level would be an important step forward to characterize inter-individual differences (*see* PROPOSAL 5). A further interesting development might consider how the development of edema around terminal vessel and airways might contribute to influence conduit resistance, thus accounting for the changes in ${\dot{V}}_{\mathrm{A}}/\dot{Q}$ distribution (*see* PROPOSAL 6).

## Clinical implications

This review presents the rather complex interaction of the factors involved in the development of lung edema; on this basis we justified that the perturbations in gas exchange occurring during exercise in healthy subjects are compatible with the development of lung edema to a various degree. The same perturbations in gas exchange are actually reported in patients being treated for pulmonary hypertension who experience dyspnea and hypoxemia as symptoms limiting their exercise tolerance. In fact, the classification of pulmonary hypertension is based on exercise tolerance/limitation (Humbert and Channick 2016; Simonneau et al. 2019).

At clinical level, besides high altitude exposure (*see* paragraph *A doubtful case: exercise at high altitude*), pulmonary hypertension is observed in apparently quite different conditions such idiopathic pulmonary fibrosis, COPD patients, interstitial pulmonary fibrosis associated with pulmonary artery fibrosis, combined pulmonary fibrosis and emphysema, common left-sided heart diseases such as aortic stenosis and left-sided heart failure. (Munson 2010). Left sided heart failure and aortic stenosis causing increased left atrial pressure and pulmonary venous pressure are rather straight forward causes of pulmonary hypertension. Yet, a basic so far unanswered pathophysiological question remains: why does pulmonary hypertension develop when associated with intrinsic lung disease? Of course pulmonary hypertension is sustained by molecular/cytokines activation causing vasoconstriction, but then again the question is why does such activation occur? A possible answer would come by hypothesizing a common pathophysiological interpretation for the different conditions based on two points emerging from this review. The first one relates with the functional interpretation of precapillary vasoconstriction in edemagenic conditions as an important mechanism aimed at avoiding an increase in alveolar capillary pressure, considered as the most edemagenic factor (*see* paragraphs *Modelling capillary flow in developing edema* and *The relative roles of the edemagenic factors*). The second point relates with the inter-individual difference in resistance/proneness to develop edema. We provide indications that subjects being more prone to develop lung edema have a greater precapillary vasoconstriction leading to a greater degree of pulmonary hypertension, a response that can be interpreted as being defensive to face a relatively higher inborn microvascular permeability (Supplementary Conceptual diagram 4). The deposition of fibrotic tissue can also be regarded as a long- term functional adaptive response to an inborn high microvascular permeability.

The treatment of the pulmonary hypertension should consider the possible consequences concerning the perturbation in lung fluid balance that strictly depends upon the state of the microvascular permeability. No surprise that administration of drugs increasing blood fluidity and microvascular flow induce lung edema (Pereda et al. 2006). Further, indications of the individual variability in response to vasodilators have been provided (Mathai et al. 2015). Moreover, safety and tolerability of vasodilator administration were found quite variable in young patients with pulmonary hypertension (Issapour et al. 2022). There is also a report of massive lung edema and death following administration of vasodilators in pulmonary hypertension (Palmer et al. 1998). Vasodilators are contraindicated in the setting of left heart failure, that implies pulmonary congestion. No danger of lung edema reasonably occurs in fibrosis, while pulmonary fibrosis combined with emphysema (Munson 2010) is a doubtful case, as emphysema is at risk of edema. The case of high altitude edema also requires caution, particularly on the hypothesis of stress failure of capillaries as in this case capillary reperfusion would make edema worse.

To summarise, the following edemagenic factors may potentially coexist in exercise (Supplementary Conceptual diagram 2 and 3):

- Capillary recruitment and increased filtration surface due to increase in cardiac output
- Increase in capillary pressure
- *Unfolding–folding* due to hyperventilation
- Increase in microvascular permeability (matrix fragmentation and increased wall shear stress)
- Waning of the *safety factors*
- Hypoxia, if present
- Intensity and duration of exercise
- Individual variability in the proneness to develop lung edema.

In humans only indirect indications can be obtained for developing interstitial lung edema.

The phase of interstitial edema (point B in Fig. 3) is clearly subclinical and can elude a diagnostic tool unable to catch ~ 10% increase in extravascular lung volume. We like to stress the point that the “*safety factors”* relating to the increase in pulmonary interstitial pressure (point B in Fig. 3) provides a lucid answer to a lucid question: “*given the sporadic nature of exercise-induced edema reports, a better question is why don’t more athletes develop alveolar flooding during exercise?*” (Hopkins 2010a). To consider of no-relevance a subclinical state of lung edema during exercise (Bates et al. 2011) might be questionable considering prevention/management of severe edema as the latter characteristically develops in a tumultuous and accelerated phase, with a time constant of a few minutes (Mazzuca et al. 2016; Parker and Townsley 2004).

## Proposals to drive possible future research

*PROPOSAL 1* The paragraph *Clinical implications* considers the challenging point of the treatment of pulmonary hypertension. The point should be stressed that the use of vasodilator drugs would vanish the anti-edemagenic role of vasoconstriction. Accordingly one should be certain that microvascular permeability is preserved. Considering the hypothesis of matrix fragmentation, it might be interesting to look for alternative drugs having a protective role on tight intercellular junctions to preserve microvascular permeability. One can quote a recent report concerning the flavonoid quercetin that, at experimental level, appears protective for the integrity of the proteoglycans of the intercellular tight junctions (Tripathi et al. 2019, 2021).

*PROPOSAL 2* One might suggest to follow the time dependent changes in impedance (*Z*_rs_)*,* resistance (*R*_rs_) and reactance (*X*_rs_) to detect the early phase of interstitial lung edema, by integrating the study with functional data (MIGET) and/or imaging data.

*PROPOSAL 3* Validate in humans the experimental approach to determine the time evolution of lung edema based on water evaporation from exhaled air.

*PROPOSAL 4* Define a calibration of imaging data in humans attempting to identify so far subclinical values of extravascular water accumulation, the threshold target value, as from the experimental approach, being (over the whole lung) a *W/D* ratio of 6.5 corresponding to a 35% increase in extravascular water.

*PROPOSAL 5* There is accumulating evidence concerning the different proneness to develop lung edema in exercise. One may further extend the knowledge concerning this point that could be of relevance not only in exercise physiology, but also in clinical medicine, including mechanical ventilation. Based on physiological parameters, at present, two approaches have been proposed. One relies on the determination of the *Vc/Dm* ratio at rest and after a subthreshold work-load in normoxia and in hypoxia (Miserocchi et al. 2022). A second method aimed at establishing the proneness to develop high acute mountain sickness relies on the estimate of the score value SHAI (severe high-altitude illness), a practical indirect method, after a work-load in hypoxia (30% of maximal power, Richalet et al. 2021).

*PROPOSAL 6* Model at biophysical level the most efficient morphology for the alveolar-capillary unit allowing to optimize the oxygen diffusive-transport mechanism and minimize microvascular fluid exchanges. Variables to be considered include geometry, permeability coefficients, capillary pressure, blood velocity, shear rate. Fractal geometry might be considered to model regional differences in fluid extravasation at capillary level; it appears stimulating to relate modifications of initial geometrical features following peri-microvascular and peri-bronchial fluid accumulation with increasing endothelial permeability. This study might help to elucidate the inter-individual differences in the proneness to develop lung edema.

## Supplementary Information

Below is the link to the electronic supplementary material.

Supplementary file1 (DOCX 387 kb)

## List of symbols

*P*_C_: Hydrostatic capillary pressure
Π_C_: Colloidosmotic capillary pressure
*J*_v_: Trans-membrane water flow
*K*_f_: Filtration coefficient
*L*_p_: Hydraulic conductance
*σ*: Proteins reflection coefficient
*P*_*i*_: Pulmonary hydrostatic interstitial pressure
Π_*i*_: Pulmonary colloidosmotic interstitial pressure
*γ*: Alveolar surface tension
*Pliq alv*: Alveolar liquid hydraulic pressure
*Πliq** alv*: Alveolar liquid colloidosmotic pressure
HS-PG: Heparan-sulphate proteoglycans
CS-PG: Chondroitin-sulphate proteoglycans
PDGL: Peptidoglycans
MIGET: Multiple Inert Gas Exchange Technique
$\dot{V}_{{\text{A}}} /\dot{Q}$: Ventilation-to-perfusion ratio
*ΔA**-a*: Alveolar-capillary O_2_ partial pressure gradient
*W/D*: Lung wet-to-dry weight ratio
$\dot{V}{\text{O}}_{2\max }$: Maximum oxygen consumption
HAPE: High Altitude Pulmonary Edema
HAPE-R: High Altitude Pulmonary Edema Resistant
HAPE-S: High Altitude Pulmonary Edema Sensitive
PAP: Pulmonary artery pressure
R5: Flow-airways resistance at 5 Hz
R20: Flow-airways resistance at 20 Hz
X5: Airways reactance at 5 Hz
LUS: Lung ultrasound
BAL: Broncho-alveolar lavage
DLCO: Carbon monoxide diffusive capacity
*D*O_2_: Oxygen diffusive capacity
*V*_c_: Pulmonary capillary blood volume
*D*_m_: Alveolar membrane diffusive capacity
$L_{{{\text{eq}}}}$: Index of alveolar-arterial O_2_ equilibration
*P*_A_: Alveolar O_2_ partial pressure
*P*_a_: Arterial O_2_ partial pressure
$P_{{\overline{v}}}$: Mixed venous O_2_ partial pressure
$$\dot{Q}$$: Cardiac output
*β*: Haemoglobin binding capacity for oxygen
*T*_t_: Average blood transit time in the pulmonary capillaries
*τ*: Time constant of the alveolar-capillary O_2_ equilibration process

## References

[CR1] Adar A, Can EY, Elma Y, Ferah MA, Kececi M, Muderrisoglu H, Akbay E, Akıncı S, Coner A, Haberal C, Cakan F, Onalan O (2022). A new and simple parameter for diagnosis pulmonary edema: Expiratory air humidity. Heart Lung.

[CR2] Agostoni E, Taglietti A, Setnikar I (1957). Absorption force of the capillaries of the visceral pleura in determination of the intrapleural pressure. Am J Physiol.

[CR3] Andersson U, Tracey KJ (2011). HMGB1 is a therapeutic target for sterile inflammation and infection. Annu Rev Immunol.

[CR4] Arborelius M, Ballidin UI, Lilja B, Lundgren CE (1972). Hemodynamic changes in man during immersion with the head above water. Aerosp Med.

[CR5] Bachofen H, Schürch S, Urbinelli M, Weibel ER (1987). Relations among alveolar surface tension, surface area, volume, and recoil pressure. J Appl Physiol (1985).

[CR6] Bachofen H, Schürch S, Weibel ER (1993). Experimental hydrostatic pulmonary edema in rabbit lungs. Barrier Lesions Am Rev Respir Dis.

[CR7] Bai C, She J, Goolaerts A, Song Y, Shen C, Shen J, Hong Q (2010). Stress failure plays a major role in the development of high-altitude pulmonary oedema in rats. Eur Respir J.

[CR8] Bartesaghi M, Beretta E, Pollastri L, Scotti V, Mandolesi G, Lanfranconi F, Miserocchi G (2014). Inter-individual differences in control of alveolar capillary blood volume in exercise and hypoxia. Respir Physiol Neurobiol.

[CR9] Bärtsch P (1999). High altitude pulmonary edema. Med Sci Sports Exerc.

[CR10] Bates ML, Farrell ET, Eldridge MW (2011). The curious question of exercise-induced pulmonary edema. Pulm Med.

[CR11] Beretta E, Lanfranconi F, Grasso GS, Bartesaghi M, Alemayehu HK, Miserocchi G (2017). Reappraisal of DLCO adjustment to interpret the adaptive response of the air-blood barrier to hypoxia. Respir Physiol Neurobiol.

[CR12] Beretta E, Lanfranconi F, Grasso GS, Bartesaghi M, Alemayehu HK, Pratali L, Catuzzo B, Giardini G, Miserocchi G (2017). Air blood barrier phenotype correlates with alveolo-capillary O2 equilibration in hypobaric hypoxia. Respir Physiol Neurobiol.

[CR13] Beretta E, Grasso GS, Forcaia G, Sancini G, Miserocchi G (2019). Differences in alveolo-capillary equilibration in healthy subjects on facing O_2_ demand. Sci Rep.

[CR14] Beretta E, Romanò F, Sancini G, Grotberg JB, Nieman GF, Miserocchi G (2021). Pulmonary interstitial matrix and lung fluid balance from normal to the acutely injured lung. Front Physiol.

[CR15] Berger MM, Hesse C, Dehnert C, Siedler H, Kleinbongard P, Bardenheuer HJ, Kelm M, Bärtsch P, Haefeli WE (2005). Hypoxia impairs systemic endothelial function in individuals prone to high-altitude pulmonary edema. Am J Respir Crit Care Med.

[CR16] Botto L, Beretta E, Daffara R, Miserocchi G, Palestini P (2006). Biochemical and morphological changes in endothelial cells in response to hypoxic interstitial edema. Respir Res.

[CR17] Botto L, Beretta E, Bulbarelli A, Rivolta I, Lettiero B, Leone BE, Miserocchi G, Palestini P (2008). Hypoxia-induced modifications in plasma membranes and lipid microdomains in A549 cells and primary human alveolar cells. J Cell Biochem.

[CR18] Brown TJ, Laurent UB, Fraser JR (1991). Turnover of hyaluronan in synovial joints: elimination of labelled hyaluronan from the knee joint of the rabbit. Exp Physiol.

[CR19] Burnham KJ, Arai TJ, Dubowitz DJ, Henderson AC, Holverda S, Buxton RB, Prisk GK, Hopkins SR (2009). Pulmonary perfusion heterogeneity is increased by sustained, heavy exercise in humans. J Appl Physiol (1985).

[CR20] Busch T, Bärtsch P, Pappert D, Grünig E, Hildebrandt W, Elser H, Falke KJ, Swenson ER (2001). Hypoxia decreases exhaled nitric oxide in mountaineers susceptible to high-altitude pulmonary edema. Am J Respir Crit Care Med.

[CR21] Buxton RB, Prisk GK, Hopkins SR (2022). A novel nonlinear analysis of blood flow dynamics applied to the human lung. J Appl Physiol (1985).

[CR22] Caillaud C, Serre-Cousiné O, Anselme F, Capdevilla X, Préfaut C (1995). Computerized tomography and pulmonary diffusing capacity in highly trained athletes after performing a triathlon. J Appl Physiol (1985).

[CR23] Canouï-Poitrine F, Veerabudun K, Larmignat P, Letournel M, Bastuji-Garin S, Richalet JP (2014). Risk prediction score for severe high altitude illness: a cohort study. PLoS ONE.

[CR24] Castagna O, Regnard J, Gempp E, Louge P, Brocq FX, Schmid B, Desruelle AV, Crunel V, Maurin A, Chopard R, MacIver DH (2018). The key roles of negative pressure breathing and exercise in the development of interstitial pulmonary edema in professional male SCUBA divers. Sports Med Open.

[CR25] Chambers RC, Laurent GJ (1996) The lung. In: Comper WD (ed) Matrix Extracellular. Tissue Function, Harwood Academic, Amsterdam, pp 378–409

[CR26] Comper WD, Laurent TC (1978). Physiological function of connective tissue polysaccharides. Physiol Rev.

[CR27] Conforti E, Fenoglio C, Bernocchi G, Bruschi O, Miserocchi GA (2002). Morpho-functional analysis of lung tissue in mild interstitial edema. Am J Physiol Lung Cell Mol Physiol.

[CR28] Cressoni M, Gallazzi E, Chiurazzi C, Marino A, Brioni M, Menga F, Cigada I, Amini M, Lemos A, Lazzerini M, Carlesso E, Cadringher P, Chiumello D, Gattinoni L (2013). Limits of normality of quantitative thoracic CT analysis. Crit Care.

[CR29] Daffara R, Botto L, Beretta E, Conforti E, Faini A, Palestini P, Miserocchi G (2004). Endothelial cells as early sensors of pulmonary interstitial edema. J Appl Physiol (1985).

[CR30] Danielli JF (1940). Capillary permeability and oedema in the perfused frog. J Physiol.

[CR31] Dehler M, Zessin E, Bärtsch P, Mairbäurl H (2006). Hypoxia causes permeability oedema in the constant-pressure perfused rat lung. Eur Respir J.

[CR32] Dellacà RL, Zannin E, Sancini G, Rivolta I, Leone BE, Pedotti A, Miserocchi G (2008). Changes in the mechanical properties of the respiratory system during the development of interstitial lung edema. Respir Res.

[CR33] Dunham-Snary KJ, Wu D, Sykes EA, Thakrar A, Parlow LRG, Mewburn JD, Parlow JL, Archer SL (2017). Hypoxic pulmonary vasoconstriction: from molecular mechanisms to medicine. Chest.

[CR34] Dunsmore SE, Rannels DE (1996). Extracellular matrix biology in the lung. Am J Physiol.

[CR35] Duplain H, Sartori C, Lepori M, Egli M, Allemann Y, Nicod P, Scherrer U (2000). Exhaled nitric oxide in high-altitude pulmonary edema: role in the regulation of pulmonary vascular tone and evidence for a role against inflammation. Am J Respir Crit Care Med.

[CR36] Edwards MR, Hunte GS, Belzberg AS, Sheel AW, Worsley DF, McKenzie DC (2000). Alveolar epithelial integrity in athletes with exercise-induced hypoxemia. J Appl Physiol (1985).

[CR37] Egan EA (1982). Lung inflation, lung solute permeability, and alveolar edema. J Appl Physiol Respir Environ Exerc Physiol.

[CR38] Eichstaedt CA, Benjamin N, Grünig E (2020). Genetics of pulmonary hypertension and high-altitude pulmonary edema. J Appl Physiol (1985).

[CR39] Eldridge MW, Dempsey JA, Haverkamp HC, Lovering AT, Hokanson JS (2004). Exercise-induced intrapulmonary arteriovenous shunting in healthy humans. J Appl Physiol (1985).

[CR40] Eldridge MW, Braun RK, Yoneda KY, Walby WF (2006). Effects of altitude and exercise on pulmonary capillary integrity: evidence for subclinical high-altitude pulmonary edema. J Appl Physiol (1985).

[CR41] Felici M, Filoche M, Sapoval B (2003). Diffusional screening in the human pulmonary acinus. J Appl Physiol (1985).

[CR42] Ferretti G, Fagoni N, Taboni A, Bruseghini P, Vinetti G (2017). The physiology of submaximal exercise: the steady state concept. Respir Physiol Neurobiol.

[CR43] Fessler JH (1957). Water and mucopolysaccharide as structural components of connective tissue. Nature.

[CR44] Fitz-Clarke JR (2007). Mechanics of airway and alveolar collapse in human breath-hold diving. Respir Physiol Neurobiol.

[CR45] Fitz-Clarke JR (2007). Computer simulation of human breath-hold diving: cardiovascular adjustments. Eur J Appl Physiol.

[CR46] Glenny RW (2011). Emergence of matched airway and vascular trees from fractal rules. J Appl Physiol (1985).

[CR47] Goldman MD, Saadeh C, Ross D (2005). Clinical applications of forced oscillation to assess peripheral airway function. Respir Physiol Neurobiol.

[CR48] Groves BM, Reeves JT, Sutton JR, Wagner PD, Cymerman A, Malconian MK, Rock PB, Young PM, Houston CS (1987). Operation Everest II: elevated high-altitude pulmonary resistance unresponsive to oxygen. J Appl Physiol (1985).

[CR49] Hamill OP, Martinac B (2001). Molecular basis of mechanotransduction in living cells. Physiol Rev.

[CR50] Hammond MD, Gale GE, Kapitan KS, Ries A, Wagner PD (1986). Pulmonary gas exchange in humans during exercise at sea level. J Appl Physiol (1985).

[CR51] Hammond MD, Gale GE, Kapitan KS, Ries A, Wagner PD (1986). Pulmonary gas exchange in humans during normobaric hypoxic exercise. J Appl Physiol (1985).

[CR52] Hanaoka M, Tanaka M, Ge RL, Droma Y, Ito A, Miyahara T, Koizumi T, Fujimoto K, Fujii T, Kobayashi T, Kubo K (2000). Hypoxia-induced pulmonary blood redistribution in subjects with a history of high-altitude pulmonary edema. Circulation.

[CR53] Hanel B, Law I, Mortensen J (2003). Maximal rowing has an acute effect on the blood-gas barrier in elite athletes. J Appl Physiol (1985).

[CR54] Hardingham TE, Fosang AJ (1992). Proteoglycans: many forms and many functions. FASEB J.

[CR55] Hårdstedt M, Seiler C, Kristiansson L, Lundeqvist D, Klingberg C, Braman Eriksson A (2020). Swimming-induced pulmonary edema: diagnostic criteria validated by lung ultrasound. Chest.

[CR56] Harf A, Pratt T, Hughes JM (1978). Regional distribution of VA/Q in man at rest and with exercise measured with krypton-81m. J Appl Physiol Respir Environ Exerc Physiol.

[CR57] Hopkins SR (2005). The lung at maximal exercise: insights from comparative physiology. Clin Chest Med.

[CR58] Hopkins SR (2010). Point: pulmonary edema does occur in human athletes performing heavy sea-level exercise. J Appl Physiol (1985).

[CR59] Hopkins SR (2010). Rebuttal from Hopkins. J Appl Physiol (1985).

[CR60] Hopkins SR (2020). Ventilation/Perfusion Relationships and Gas Exchange: Measurement Approaches. Compr Physiol.

[CR61] Hopkins SR, Harms CA (2004). Gender and pulmonary gas exchange during exercise. Exerc Sport Sci Rev.

[CR62] Hopkins SR, Belzberg AS, Wiggs BR, McKenzie DC (1996). Pulmonary transit time and diffusion limitation during heavy exercise in athletes. Respir Physiol.

[CR63] Hopkins SR, Schoene RB, Henderson WR, Spragg RG, Martin TR, West JB (1997). Intense exercise impairs the integrity of the pulmonary blood-gas barrier in elite athletes. Am J Respir Crit Care Med.

[CR64] Hopkins SR, Gavin TP, Siafakas NM, Haseler LJ, Olfert IM, Wagner H, Wagner PD (1998). Effect of prolonged, heavy exercise on pulmonary gas exchange in athletes. J Appl Physiol (1985).

[CR65] Hopkins SR, Schoene RB, Henderson WR, Spragg RG, West JB (1998). Sustained submaximal exercise does not alter the integrity of the lung blood-gas barrier in elite athletes. J Appl Physiol (1985).

[CR66] Hopkins SR, Olfert IM, Wagner PD (2009). Point: exercise-induced intrapulmonary shunting is imaginary. J Appl Physiol (1985).

[CR67] Hopkins SR, Wielpütz MO, Kauczor HU (2012). Imaging lung perfusion. J Appl Physiol (1985).

[CR68] Hultgren HN, Lopez CE, Lundberg E, Miller H (1964). Physiologic studies of pulmonary edema at high altitude. Circulation.

[CR69] Humbert M, Channick RN (2016). Pulmonary hypertension. Curr Opin Pulm Med.

[CR70] Ingber DE (2003). Tensegrity II. How structural networks influence cellular information processing networks. J Cell Sci.

[CR71] Issapour A, Frank B, Crook S, Hite MD, Dorn ML, Rosenzweig EB, Ivy DD, Krishnan US (2022). Safety and tolerability of combination therapy with ambrisentan and tadalafil for the treatment of pulmonary arterial hypertension in children: Real-world experience. Pediatr Pulmonol.

[CR72] Ito S (1969). Structure and function of the glycocalyx. Fed Proc.

[CR73] Jin J, Fang F, Gao W, Chen H, Wen J, Wen X, Chen J (2021). The structure and function of the glycocalyx and its connection with blood-brain barrier. Front Cell Neurosci.

[CR74] Kedem O, Katchalsky A (1958). Thermodynamic analysis of the permeability of biological membranes to non-electrolytes. Biochim Biophys Acta.

[CR75] Knudsen L, Ochs M (2018). The micromechanics of lung alveoli: structure and function of surfactant and tissue components. Histochem Cell Biol.

[CR76] Landis EM (1934). Capillary pressure and capillary permeability. Physiol Rev.

[CR77] Laurent TC, Fraser JR (1992). Hyaluronan. FASEB J.

[CR78] Lovering AT (2010). The exercise-induced pulmonary edema story doesn’t hold water. J Appl Physiol (1985).

[CR79] Lovering AT, Romer LM, Haverkamp HC, Pegelow DF, Hokanson JS, Eldridge MW (2008). Intrapulmonary shunting and pulmonary gas exchange during normoxic and hypoxic exercise in healthy humans. J Appl Physiol (1985).

[CR80] Lovering AT, Haverkamp HC, Romer LM, Hokanson JS, Eldridge MW (2009). Transpulmonary passage of 99mTc macroaggregated albumin in healthy humans at rest and during maximal exercise. J Appl Physiol (1985).

[CR81] Luks AM, Robertson HT, Swenson ER (2007). An ultracyclist with pulmonary edema during the Bicycle Race Across America. Med Sci Sports Exerc.

[CR82] Maggiorini M, Mélot C, Pierre S, Pfeiffer F, Greve I, Sartori C, Lepori M, Hauser M, Scherrer U, Naeije R (2001). High-altitude pulmonary edema is initially caused by an increase in capillary pressure. Circulation.

[CR83] Manohar M (1993). Pulmonary artery wedge pressure increases with high-intensity exercise in horses. Am J Vet Res.

[CR84] Manohar M (1994). Pulmonary vascular pressures of thoroughbreds increase rapidly and to a higher level with rapid onset of high-intensity exercise than slow onset. Equine Vet J.

[CR85] Mansour E, Vishinkin R, Rihet S, Saliba W, Fish F, Sarfati P, Haick H (2019). Measurement of temperature and relative humidity in exhaled breath. Sens Actuators, B Chem.

[CR86] Mathai SC, Hassoun PM, Puhan MA, Zhou Y, Wise RA (2015). Sex differences in response to tadalafil in pulmonary arterial hypertension. Chest.

[CR87] Mazzuca E, Aliverti A, Miserocchi G (2016). Computational micro-scale model of control of extravascular water and capillary perfusion in the air blood barrier. J Theor Biol.

[CR88] Mazzuca E, Aliverti A, Miserocchi G (2019). Understanding Vasomotion of Lung Microcirculation by In Vivo Imaging. J Imaging.

[CR89] McKechnie JK, Leary WP, Noakes TD, Kallmeyer JC, MacSearraigh ET, Olivier LR (1979). Acute pulmonary oedema in two athletes during a 90-km running race. S Afr Med J.

[CR90] McKenzie DC, O'Hare TJ, Mayo J (2005). The effect of sustained heavy exercise on the development of pulmonary edema in trained male cyclists. Respir Physiol Neurobiol.

[CR91] Meyer BJ, Meyer A, Guyton AC (1968). Interstitial fluid pressure. V. Negative pressure in the lungs. Circ Res.

[CR92] Michel CC, Phillips ME (1987). Steady-state fluid filtration at different capillary pressures in perfused frog mesenteric capillaries. J Physiol.

[CR93] Miserocchi G (2009). Mechanisms controlling the volume of pleural fluid and extravascular lung water. Eur Respir Rev.

[CR94] Miserocchi G, Rivolta I (2012). Mechanistic considerations on the development of lung edema: vascular, perivascular and molecular aspects from early stage to tissue and vascular remodeling stage. Curr Respir Med Rev.

[CR95] Miserocchi G, Negrini D, Mukenge S, Turconi P, Del Fabbro M (1989). Liquid drainage through the peritoneal diaphragmatic surface. J Appl Physiol (1985).

[CR96] Miserocchi G, Negrini D, Gonano C (1990). Direct measurement of interstitial pulmonary pressure in in situ lung with intact pleural space. J Appl Physiol (1985).

[CR97] Miserocchi G, Negrini D, Gonano C (1991). Parenchymal stress affects interstitial and pleural pressures in in situ lung. J Appl Physiol (1985).

[CR98] Miserocchi G, Negrini D, Del Fabbro M, Venturoli D (1993). Pulmonary interstitial pressure in intact in situ lung: transition to interstitial edema. J Appl Physiol (1985).

[CR99] Miserocchi G, Venturoli D, Negrini D, Del Fabbro M (1993). Model of pleural fluid turnover. J Appl Physiol (1985).

[CR100] Miserocchi G, Passi A, Negrini D, Del Fabbro M, De Luca G (2001). Pulmonary interstitial pressure and tissue matrix structure in acute hypoxia. Am J Physiol Lung Cell Mol Physiol.

[CR101] Miserocchi G, Negrini D, Passi A, De Luca G (2001). Development of lung edema: interstitial fluid dynamics and molecular structure. News Physiol Sci.

[CR102] Miserocchi G, Messinesi G, Tana F, Passoni E, Adamo S, Romano R, Beretta E (2008). Mechanisms behind inter-individual differences in lung diffusing capacity. Eur J Appl Physiol.

[CR103] Miserocchi G, Beretta E, Rivolta I, Bartesaghi M (2022). Role of the air-blood barrier phenotype in lung oxygen uptake and control of extravascular water. Front Physiol.

[CR104] Mitzner W, Sylvester JT (1986). Lymph flow and lung weight in isolated sheep lungs. J Appl Physiol (1985).

[CR105] Morris CE, Homann U (2001). Cell surface area regulation and membrane tension. J Membr Biol.

[CR106] Moudgil R, Michelakis ED, Archer SL (2005). Hypoxic pulmonary vasoconstriction. J Appl Physiol (1985).

[CR107] Munson JC (2010). Combined pulmonary fibrosis and emphysema: a high-pressure situation. Eur Respir J.

[CR108] Negrini D (1995). Pulmonary microvascular pressure profile during development of hydrostatic edema. Microcirculation.

[CR109] Negrini D, Gonano C, Miserocchi G (1992). Microvascular pressure profile in intact in situ lung. J Appl Physiol (1985).

[CR110] Negrini D, Passi A, de Luca G, Miserocchi G (1996). Pulmonary interstitial pressure and proteoglycans during development of pulmonary edema. Am J Physiol.

[CR111] Negrini D, Passi A, De Luca G, Miserochi G (1998). Proteoglycan involvement during development of lesional pulmonary edema. Am J Physiol.

[CR112] Negrini D, Passi A, Bertin K, Bosi F, Wiig H (2001). Isolation of pulmonary interstitial fluid in rabbits by a modified wick technique. Am J Physiol Lung Cell Mol Physiol.

[CR113] Nowadly CD, Kelley KM, Crane DH, Rose JS (2021). Evaluation of high altitude interstitial pulmonary edema in healthy participants using rapid 4-view lung ultrasound protocol during staged ascent to everest base camp. Wilderness Environ Med.

[CR114] Palestini P, Botto L, Rivolta I, Miserocchi G (2011). Remodelling of membrane rafts expression in lung cells as an early sign of mechanotransduction-signalling in pulmonary edema. J Lipids.

[CR115] Palmer SM, Robinson LJ, Wang A, Gossage JR, Bashore T, Tapson VF (1998). Massive pulmonary edema and death after prostacyclin infusion in a patient with pulmonary veno-occlusive disease. Chest.

[CR116] Parker JC (2007). Hydraulic conductance of lung endothelial phenotypes and Starling safety factors against edema. Am J Physiol Lung Cell Mol Physiol.

[CR117] Parker JC, Townsley MI (2004). Evaluation of lung injury in rats and mice. Am J Physiol Lung Cell Mol Physiol.

[CR118] Passi A, Negrini D, Albertini R, Miserocchi G, De Luca G (1999). The sensitivity of versican from rabbit lung to gelatinase A (MMP-2) and B (MMP-9) and its involvement in the development of hydraulic lung edema. FEBS Lett.

[CR119] Pereda J, Gómez-Cambronero L, Alberola A, Fabregat G, Cerdá M, Escobar J, Sabater L, García-de-la-Asunción J, Viña J, Sastre J (2006). Co-administration of pentoxifylline and thiopental causes death by acute pulmonary oedema in rats. Br J Pharmacol.

[CR120] Picano E, Pellikka PA (2016). Ultrasound of extravascular lung water: a new standard for pulmonary congestion. Eur Heart J.

[CR121] Piiper J, Scheid P (1981). Model for capillary-alveolar equilibration with special reference to O2 uptake in hypoxia. Respir Physiol.

[CR122] Podolsky A, Eldridge MW, Richardson RS, Knight DR, Johnson EC, Hopkins SR, Johnson DH, Michimata H, Grassi B, Feiner J, Kurdak SS, Bickler PE, Severinghaus JW, Wagner PD (1996). Exercise-induced VA/Q inequality in subjects with prior high-altitude pulmonary edema. J Appl Physiol (1985).

[CR123] Reitsma S, Slaaf DW, Vink H, van Zandvoort MA, oude Egbrink MG (2007). The endothelial glycocalyx: composition, functions, and visualization. Pflugers Arch.

[CR124] Richalet JP, Larmignat P, Poitrine E, Letournel M, Canouï-Poitrine F (2012). Physiological risk factors for severe high-altitude illness: a prospective cohort study. Am J Respir Crit Care Med.

[CR125] Richalet JP, Pillard F, Moal LED, Rivière D, Oriol P, Poussel M, Chenuel B, Doutreleau S, Vergès S, Demanez S, Vergnion M, Boulet JM, Douard H, Dupré M, Mesland O, Remetter R, Lonsdorfer-Wolf E, Frey A, Vilcoq L, Nedelec Jaffuel A, Debeaumont D, Duperrex G, Lecoq F, Hédon C, Hayot M, Giardini G, Lhuissier FJ (2021). Validation of a score for the detection of subjects with high risk for severe high-altitude illness. Med Sci Sports Exerc.

[CR126] Rivolta I, Lucchini V, Rocchetti M, Kolar F, Palazzo F, Zaza A, Miserocchi G (2011). Interstitial pressure and lung oedema in chronic hypoxia. Eur Respir J.

[CR127] Roberts CR, Wight TN, Hascall VC, Crystal RG, West JB, Weibel E, Barnes P (1997). Proteoglycans. The Lung: Scientific Foundations.

[CR128] Sá RC, Henderson AC, Simonson T, Arai TJ, Wagner H, Theilmann RJ, Wagner PD, Prisk GK, Hopkins SR (2017). Measurement of the distribution of ventilation-perfusion ratios in the human lung with proton MRI: comparison with the multiple inert-gas elimination technique. J Appl Physiol (1985).

[CR129] Sabbadini M, Barisani D, Conforti E, Marozzi A, Ginelli E, Miserocchi G, Meneveri R (2003). Gene expression analysis in interstitial lung edema induced by saline infusion. Biochim Biophys Acta.

[CR130] Sapoval B (1993) Transfer to and across irregular membranes modelled by fractal geometry. In: Nonnenmacher TF, Losa GA, Weibel ER (eds) Fractals in biology and medicine. Mathematics and biosciences in interaction. Birkhäuser, Basel, pp 241–250. 10.1007/978-3-0348-8501-0_21

[CR131] Schaffartzik W, Poole DC, Derion T, Tsukimoto K, Hogan MC, Arcos JP, Bebout DE, Wagner PD (1992). VA/Q distribution during heavy exercise and recovery in humans: implications for pulmonary edema. J Appl Physiol (1985).

[CR132] Scherrer U, Vollenweider L, Delabays A, Savcic M, Eichenberger U, Kleger GR, Fikrle A, Ballmer PE, Nicod P, Bärtsch P (1996). Inhaled nitric oxide for high-altitude pulmonary edema. N Engl J Med.

[CR133] Schoene RB, Hackett PH, Henderson WR, Sage EH, Chow M, Roach RC, Mills WJ, Martin TR (1986). High-altitude pulmonary edema. Characteristics of lung lavage fluid. JAMA.

[CR134] Schoene RB, Swenson ER, Pizzo CJ, Hackett PH, Roach RC, Mills WJ, Henderson WR, Martin TR (1988). The lung at high altitude: bronchoalveolar lavage in acute mountain sickness and pulmonary edema. J Appl Physiol (1985).

[CR135] Schumacher YO, Ahlgrim C, Prettin S, Pottgiesser T (2011). Physiology, power output, and racing strategy of a Race Across America finisher. Med Sci Sports Exerc.

[CR136] Sheel AW, McKenzie DC (2010). Counterpoint: Pulmonary edema does not occur in human athletes performing heavy sea-level exercise. J Appl Physiol (1985).

[CR137] Simonneau G, Montani D, Celermajer DS, Denton CP, Gatzoulis MA, Krowka M, Williams PG, Souza R (2019). Haemodynamic definitions and updated clinical classification of pulmonary hypertension. Eur Respir J.

[CR138] Starling EH (1896). On the absorption of fluids from the connective tissue spaces. J Physiol.

[CR139] Starling EH, Tubby AH (1894). On Absorption from and Secretion into the Serous Cavities. J Physiol.

[CR140] Stickland MK, Tedjasaputra V, Seaman C, Fuhr DP, Collins SÉ, Wagner H, van Diepen S, Byers BW, Wagner PD, Hopkins SR (2019). Intra-pulmonary arteriovenous anastomoses and pulmonary gas exchange: evaluation by microspheres, contrast echocardiography and inert gas elimination. J Physiol.

[CR141] Swenson ER, Bärtsch P (2012). High-altitude pulmonary edema. Compr Physiol.

[CR142] Swenson ER, Maggiorini M, Mongovin S, Gibbs JS, Greve I, Mairbäurl H, Bärtsch P (2002). Pathogenesis of high-altitude pulmonary edema: inflammation is not an etiologic factor. JAMA.

[CR143] Swenson KE, Berger MM, Sareban M, Macholz F, Schmidt P, Schiefer LM, Mairbäurl H, Swenson ER (2020). Rapid ascent to 4559 m Is associated with increased plasma components of the vascular endothelial glycocalyx and may be associated with acute mountain sickness. High Alt Med Biol.

[CR144] Sydykov A, Mamazhakypov A, Maripov A, Kosanovic D, Weissmann N, Ghofrani HA, Sarybaev AS, Schermuly RT (2021). Pulmonary hypertension in acute and chronic high altitude maladaptation disorders. Int J Environ Res Public Health.

[CR145] Tarbell JM (2010). Shear stress and the endothelial transport barrier. Cardiovasc Res.

[CR146] Tarbell JM, Demaio L, Zaw MM (1999). Effect of pressure on hydraulic conductivity of endothelial monolayers: role of endothelial cleft shear stress. J Appl Physiol (1985).

[CR147] Taylor AE, Parker JC, Fishman AP, Fisher AB (1985). The interstitial spaces and lymph flow. Handbook of physiology. The respiratory system. Circulation and non respiratory function.

[CR148] Tedjasaputra V, Sá RC, Anderson KM, Prisk GK (2019). Heavy upright exercise increases ventilation-perfusion mismatch in the basal lung: indirect evidence for interstitial pulmonary edema. J Appl Physiol (1985).

[CR149] Tripathi A, Kumar B, Sagi SSK (2019). Prophylactic efficacy of Quercetin in ameliorating the hypoxia induced vascular leakage in lungs of rats. PLoS ONE.

[CR150] Tripathi A, Hazari PP, Mishra AK, Kumar B, Sagi SSK (2021). Quercetin: a savior of alveolar barrier integrity under hypoxic microenvironment. Tissue Barriers.

[CR151] Unruh HW, Goldberg HS, Oppenheimer L (1984). Pulmonary interstitial compartments and tissue resistance to fluid flux. J Appl Physiol Respir Environ Exerc Physiol.

[CR152] Wagner PD, Gale GE, Moon RE, Torre-Bueno JR, Stolp BW, Saltzman HA (1986). Pulmonary gas exchange in humans exercising at sea level and simulated altitude. J Appl Physiol (1985).

[CR153] Wagner PD, Sutton JR, Reeves JT, Cymerman A, Groves BM, Malconian MK (1987). Operation Everest II: pulmonary gas exchange during a simulated ascent of Mt. Everest. J Appl Physiol (1985).

[CR154] Wagner PD, Gillespie JR, Landgren GL, Fedde MR, Jones BW, DeBowes RM, Pieschl RL, Erickson HH (1989). Mechanism of exercise-induced hypoxemia in horses. J Appl Physiol (1985).

[CR155] Weibel ER (1973). Morphological basis of alveolar-capillary gas exchange. Physiol Rev.

[CR156] Weibel ER (2015). On the tricks alveolar epithelial cells play to make a good lung. Am J Respir Crit Care Med.

[CR157] Weibel ER, Knight BW (1964). A morphometric study on the thickness of the pulmonary air-blood barrier. J Cell Biol.

[CR158] Weinbaum S, Tarbell JM, Damiano ER (2007). The structure and function of the endothelial glycocalyx layer. Annu Rev Biomed Eng.

[CR159] West JB, Tsukimoto K, Mathieu-Costello O, Prediletto R (1991). Stress failure in pulmonary capillaries. J Appl Physiol.

[CR160] Whitwell KE, Greet TR (1984). Collection and evaluation of tracheobronchial washes in the horse. Equine Vet J.

[CR161] Wu DX, Weibel ER, Bachofen H, Schürch S (1995). Lung lesions in experimental hydrostatic pulmonary edema: an electron microscopic and morphometric study. Exp Lung Res.

[CR162] Yang H, Wang H, Andersson U (2020). Targeting inflammation driven by HMGB1. Front Immunol.

[CR163] Yokoyama T, Farhi LE (1967). Study of ventilation-perfusion ratio distribution in the anesthetized dog by multiple inert gas washout. Respir Physiol.

[CR164] Yoshikawa S, King JA, Lausch RN, Penton AM, Eyal FG, Parker JC (2004). Acute ventilator-induced vascular permeability and cytokine responses in isolated and in situ mouse lungs. J Appl Physiol (1985).

[CR165] Zavorsky GS (2007). Evidence of pulmonary oedema triggered by exercise in healthy humans and detected with various imaging techniques. Acta Physiol (oxf).

[CR166] Zavorsky GS, Milne EN, Lavorini F, Rienzi JP, Lavin KM, Straub AM, Pistolesi M (2014). Interstitial lung edema triggered by marathon running. Respir Physiol Neurobiol.

