# Supplementary material for: A century of exercise physiology: lung fluid balance during and following exercise
Source: Eur J Appl Physiol. 2022 Oct 20;123(1):1–24. doi: 10.1007/s00421-022-05066-3 (PMC9813172; doi:10.1007/s00421-022-05066-3)
Supplement: Supplementary file 1 — Supplementary file1 (DOCX 387 kb) [file 421_2022_5066_MOESM1_ESM.docx]

**Conceptual Diagrams**

1. Fluid balance in physiological condition


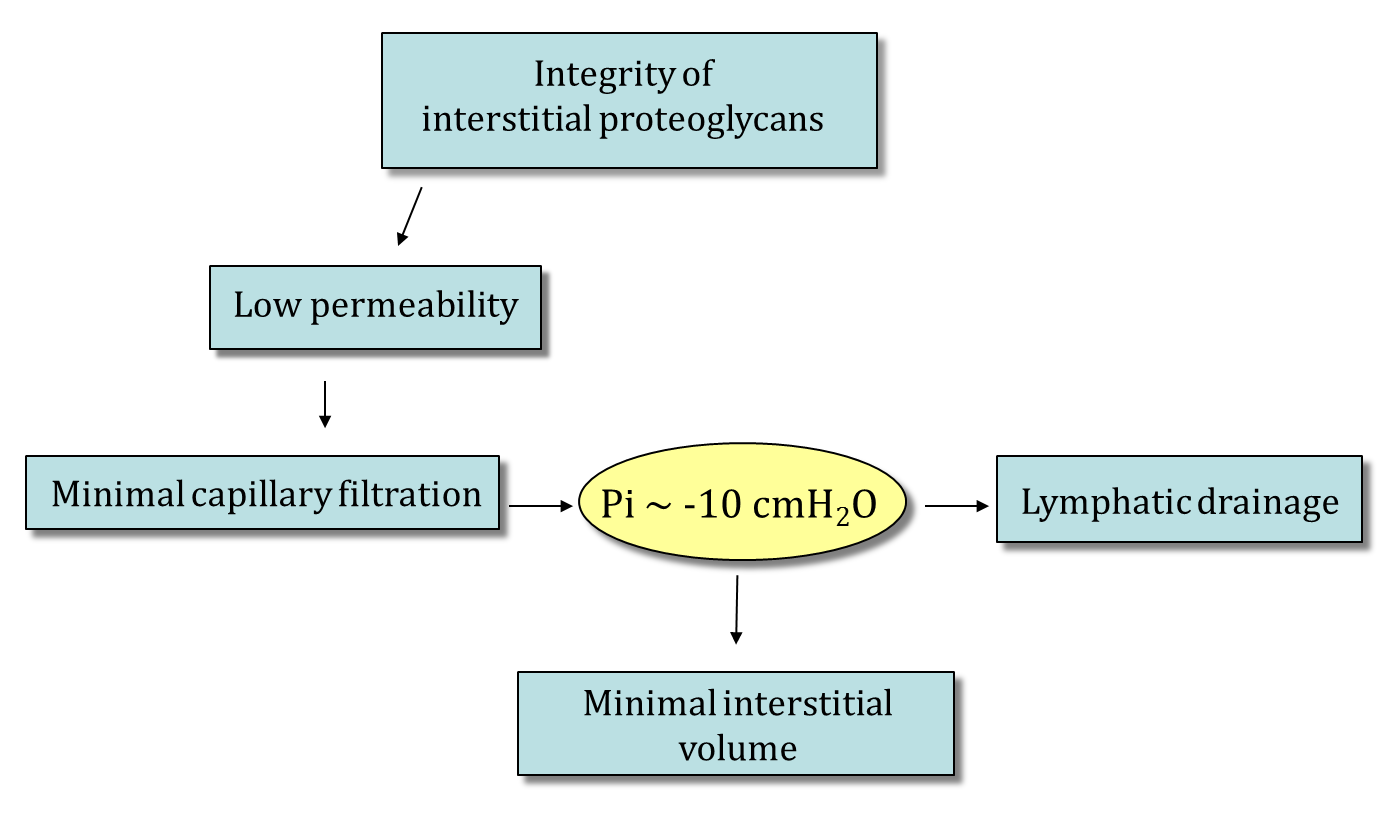


2. Facing an increase in fluid filtration: «a safety factor»


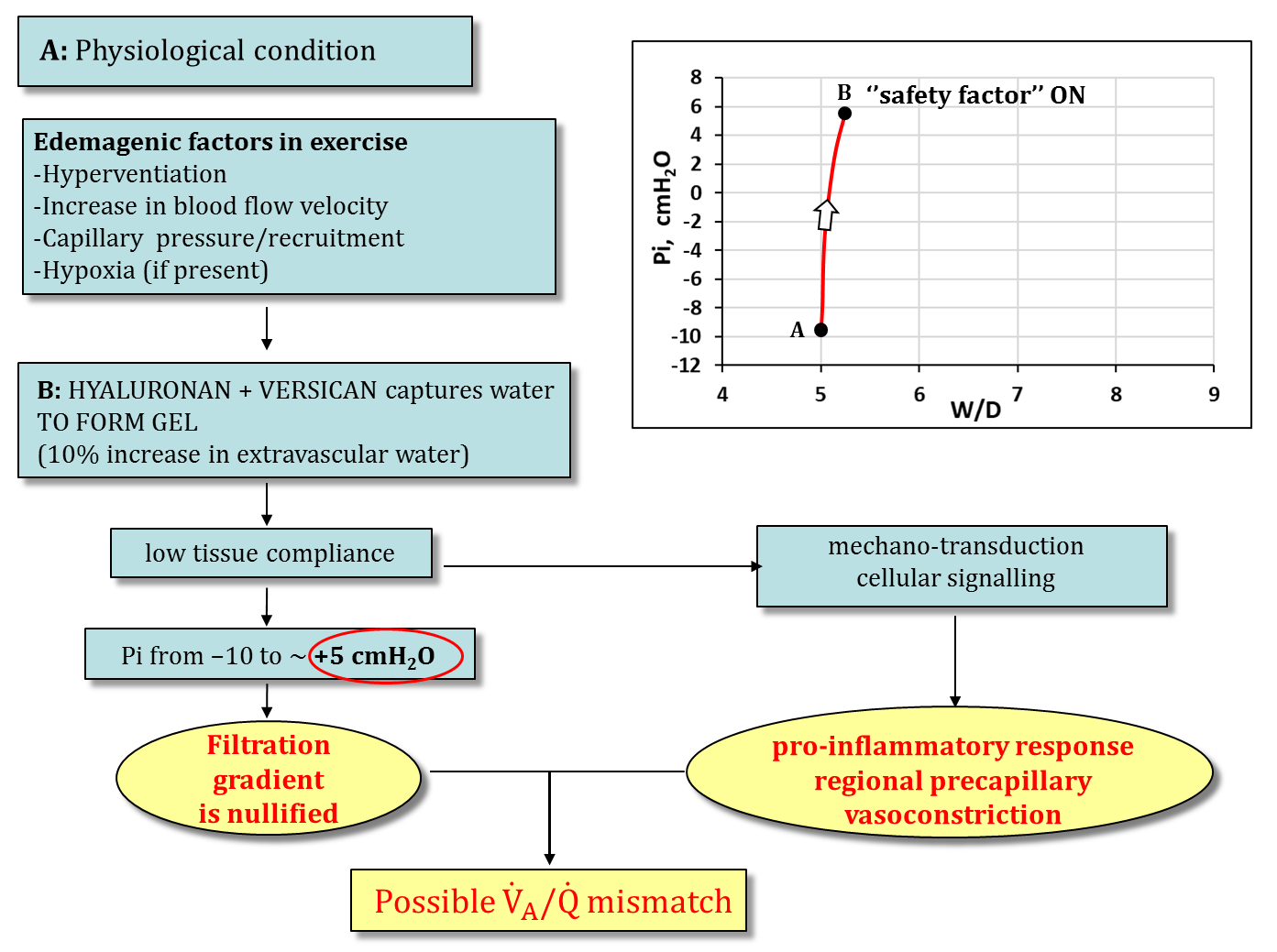


3. The aggravation of lung edema in exercise


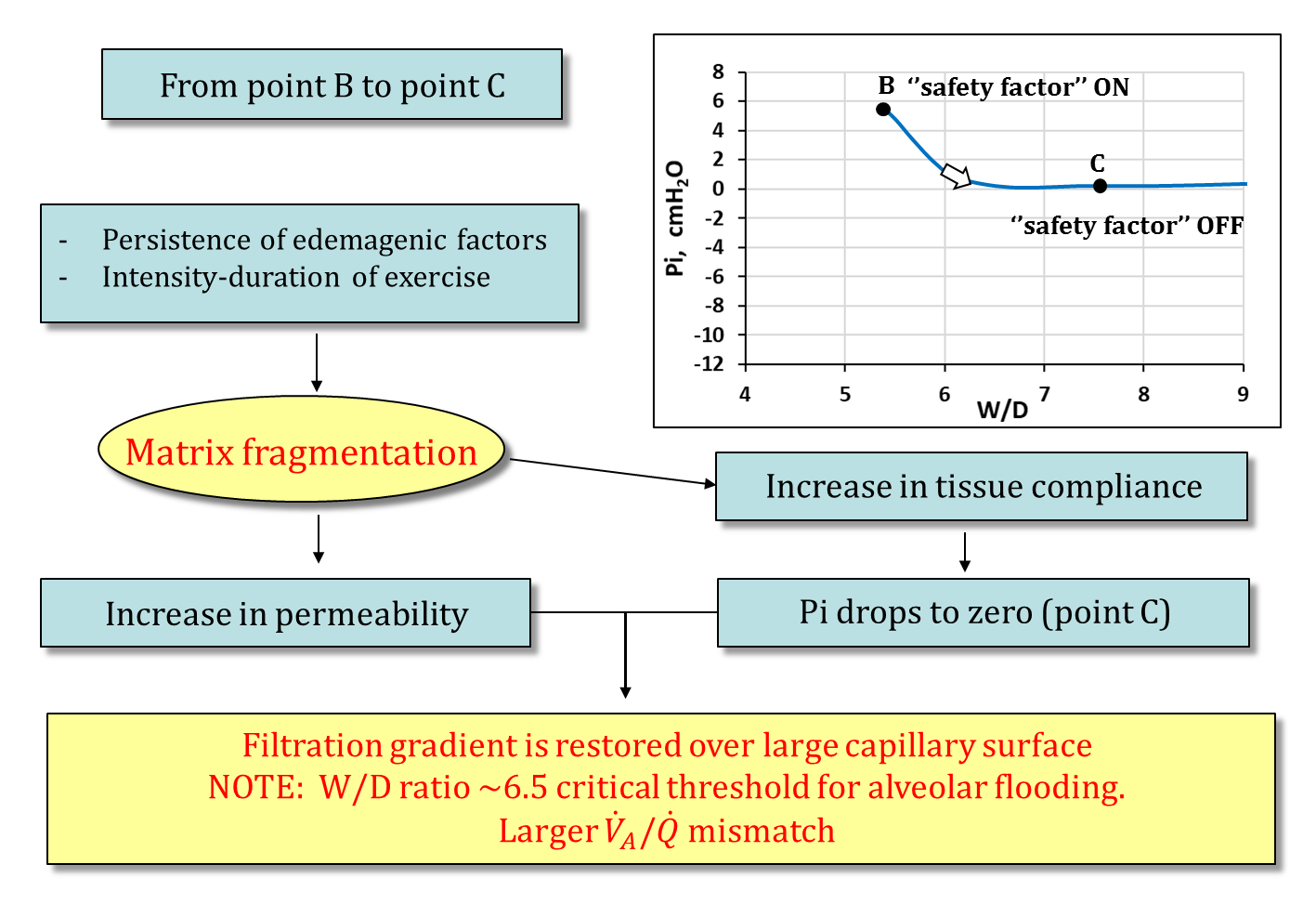


4. Proneness to develop lung edema

**
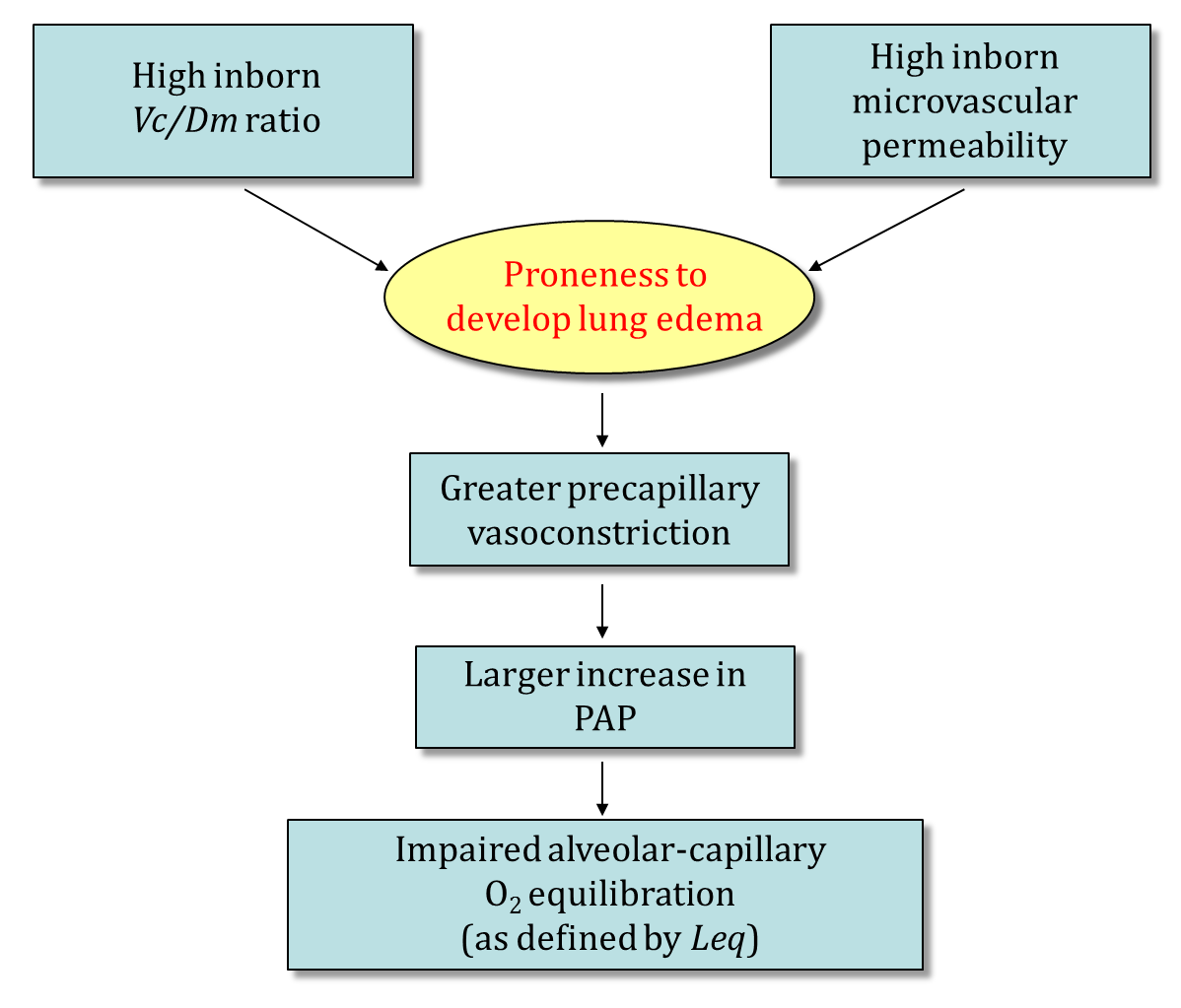
**
